# DockOpt: A Tool for Automatic Optimization of Docking Models

**DOI:** 10.1021/acs.jcim.3c01406

**Published:** 2024-01-11

**Authors:** Ian S. Knight, Olivier Mailhot, Khanh G. Tang, John J. Irwin

**Affiliations:** Department of Pharmaceutical Chemistry, UCSF, 1700 Fourth Street, San Francisco, California 94158-2330, United States

## Abstract

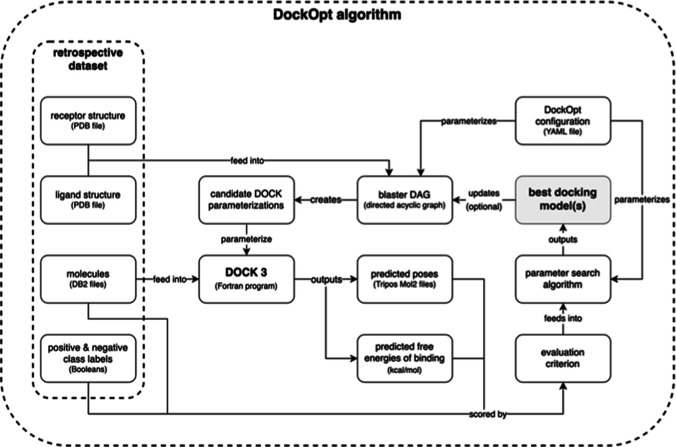

Molecular docking is a widely used technique for leveraging protein structure for ligand discovery, but it remains difficult to utilize due to limitations that have not been adequately addressed. Despite some progress toward automation, docking still requires expert guidance, hindering its adoption by a broader range of investigators. To make docking more accessible, we developed a new utility called DockOpt, which automates the creation, evaluation, and optimization of docking models prior to their deployment in large-scale prospective screens. DockOpt outperforms our previous automated pipeline across all 43 targets in the DUDE-Z benchmark data set, and the generated models for 84% of targets demonstrate sufficient enrichment to warrant their use in prospective screens, with normalized LogAUC values of at least 15%. DockOpt is available as part of the Python package Pydock3 included in the UCSF DOCK 3.8 distribution, which is available for free to academic researchers at https://dock.compbio.ucsf.edu and free for everyone upon registration at https://tldr.docking.org.

## Introduction

Molecular docking is widely used for ligand discovery, both in industry and academia.^1−3^ The goal of docking is to predict the binding affinity and pose of small molecules in the binding site of a target protein. The method can screen libraries of billions of molecules and, unlike ligand-based methods, often discovers novel ligands entirely unrelated to those previously known.^2,4−13^ In some cases, docking can lead to the discovery of compounds in the sub-nM range,^4,5,8,10^ with some of these being active *in vivo*.^5,8−10^ However, compared to other techniques in computational biology, such as homology modeling^14−16^ and sequence database searching,^17^ docking as a procedure remains labor-intensive and intimidating to new users, thereby limiting its wider adoption and hindering its application on a proteomic scale. Docking software is typically complicated and comes with a steep learning curve, making it difficult to use to its full potential. This is especially true during the model optimization stage of the docking process, which involves fine-tuning numerous parameters of the model to improve its accuracy and reliability. It does not help that even when performed by experts, docking can still sometimes fail to accurately reproduce experimentally determined binding characteristics for some targets. These liabilities have diminished the technique’s overall impact, not only by deterring researchers coming from limited computational backgrounds but also by making it arduous even for experienced computational researchers to deploy docking models at a large scale on the order of billions of molecules.

Automating the several stages of the docking process all in a single pipeline could significantly reduce the need for expert involvement, which would enhance the accessibility of docking as a technology. An ideal pipeline would simplify the preparation of the docking model for those with less experience while still allowing experts the option to adjust the model as needed. Moreover, beyond merely creating a docking model, an optimal pipeline would also optimize the model’s parameters to ensure that its performance is at least comparable to that of a model produced by an expert provided the same initial data. For this to be possible, the pipeline must first be capable of evaluating the quality of the candidate models. Typically, this evaluation is performed using retrospective docking.^18^ This method involves assessing a model’s ability to (1) accurately predict the binding characteristics of known ligand structures, such as pose and (2) consistently assign them more favorable docking scores than those of designated decoy molecules. These decoy molecules may be property-matched to the known ligands or selected by other methods.^19^

Several attempts have been made to automate some parts of the docking process over the past 14 years,^20−22^ a few of which have web interfaces.^23−27^ While many of these computational pipelines excel at automating the routine aspects of generating docking models, they usually lack the capability to integrate the nuanced practices of evaluation and optimization that experts commonly apply in the preparation of models for large-scale screening.^18,28−31^ As both evaluation and optimization are essential for developing models that can reliably distinguish between binding and nonbinding compounds,^18^ integrating them into these pipelines represents a crucial milestone toward automating the specialized skills of docking experts.

Our work on automating the docking process began in 2009 with the introduction of the web-based tool *DOCK Blaster*.^20^ Although it successfully performed retrospective docking on thousands of targets, DOCK Blaster had noteworthy limitations. For instance, it lacked a framework for evaluating results, making it difficult to trust the predicted binding modes of resultant models without further assessment. Consequently, it was also unable to optimize the parameters of the DOCK scoring function, which estimates the binding affinity between a candidate molecule pose and the target protein. In effect, DOCK Blaster served merely as a prototype composed of isolated scripts that made it fragile and difficult to maintain or develop further. In short, although DOCK Blaster demonstrated potential, its shortcomings highlighted the need for a more robust automated pipeline.

Since the appearance of DOCK Blaster, several other web-based docking pipelines have appeared,^25,32−35^ some designed with the scalability of the cloud in mind.^36−38^ There have also been many reports of increasingly automated docking software without web interfaces.^6,38−47^

Given these limitations, we focused our efforts on improving our own techniques to streamline the docking process. To that end, we rewrote the command line tool for creating docking models, Blastermaster, making it more modular and feature-rich,^48^ and standardized and published our lab’s docking protocol.^18^ Despite these advancements, expert supervision remained necessary for conducting model evaluation and optimization, and the absence of a web interface curtailed the potential for wider accessibility to these improvements.

To address these challenges, we introduce DockOpt, a new automated docking pipeline that enables the creation, evaluation, and optimization of docking models using a single tool. DockOpt is part of the Python package Pydock3, a toolkit dedicated to the standardization and enhancement of docking methodologies, specifically designed to complement UCSF DOCK 3.8 and subsequent versions. To evaluate the utility of DockOpt, we benchmarked it against the DUDE-Z data set.^19^

## Methods

The Python package Pydock3 is part of the DOCK 3.8 software distribution and is compatible with python ≥3.8.1, <3.11. DOCK 3.8 is compatible with modern Linux operating systems. The scripts DockOpt and Blastermaster are included with Pydock3, and all dependencies are defined in the pyproject.toml file.^49^

### Transitioning from Blastermaster to DockOpt

Blastermaster^48^ is a command line tool that generates docking models for protein target binding sites. DockOpt builds upon Blastermaster by creating multiple models in a single pass and then optimizing parameters based on the retrospective docking performance observed for these models. DockOpt evaluates models using a specified criterion, such as normalized LogAUC (also called the “enrichment score”).^50^ To efficiently evaluate many candidate models, DockOpt employs a designated job scheduler (*e.g.*, Slurm) to test several models in parallel. In summary, DockOpt enhances the functionality of Blastermaster by integrating model evaluation and concurrent optimization into the process of generating docking models.

### Creating Docking Models with DockOpt

**Figure 1:**
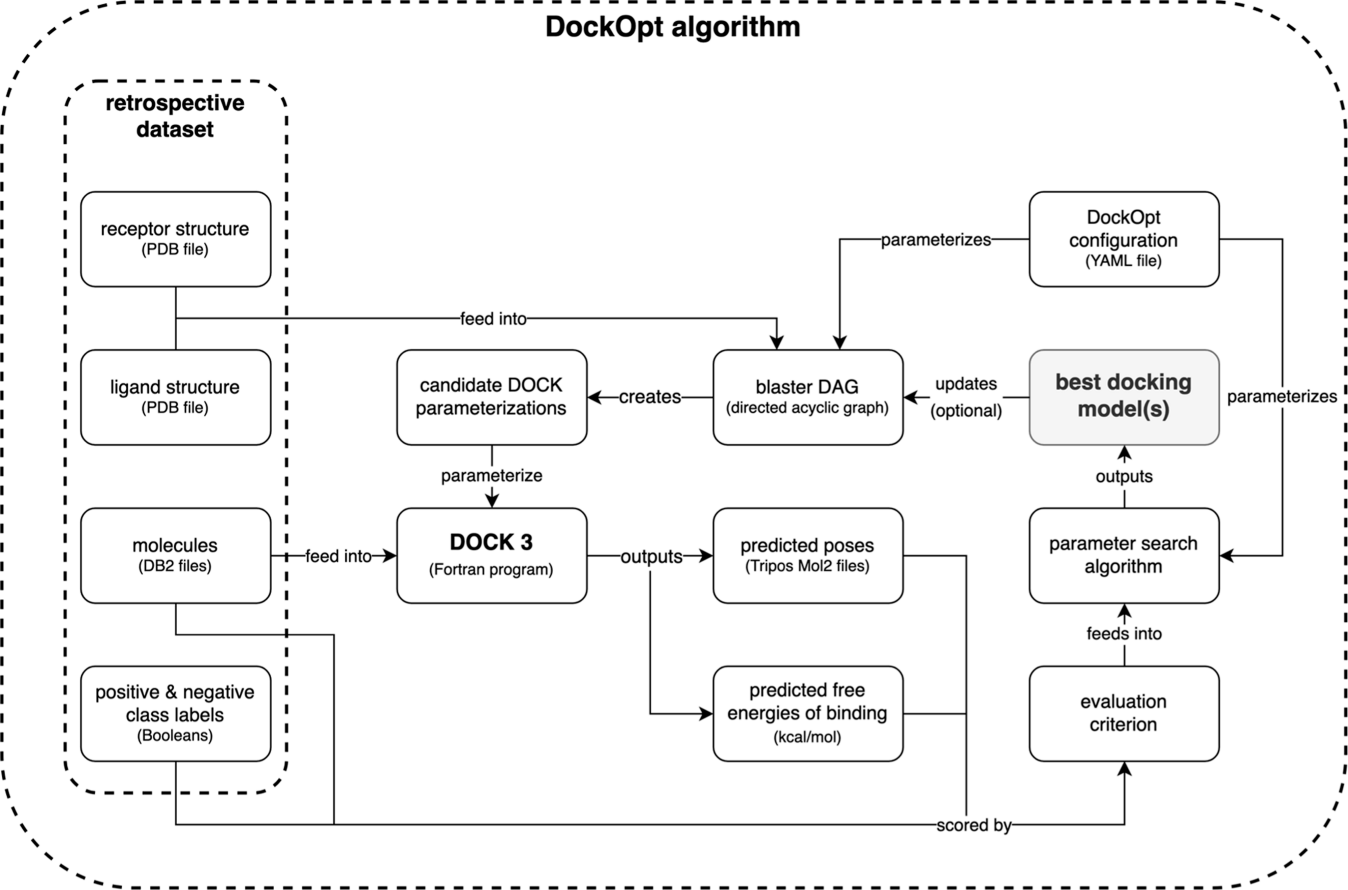
Schematic representation of the DockOpt algorithm. The retrospective data set provided by the user consists of (1) a receptor structure, (2) a ligand structure, (3) positive-class molecules (*e.g.*, known ligands), and (4) negative-class molecules (*e.g.*, property-matched decoys). The parameters in the dockopt_config.yaml file determine the structure of the *blaster directed acyclic graph* (*blaster DAG*), which takes the receptor and ligand structures as input and produces all candidate DOCK parameterizations as output. The blaster DAG is so-called because it uses the subroutines of Blastermaster to create DOCK parameterizations. Each resultant parameterization modifies the DOCK program as a unique docking model. All the models are used to dock the provided molecules, each labeled as belonging to either the positive class or the negative class. The output docking scores and poses are used by the specified criteria to evaluate the parameterizations, which are then ranked accordingly. At this point, the parameters in the dockopt_config.yaml file determine (1) whether the program should iterate and, if so, (2) what proportion of top parameterizations to advance to the next iteration, (3) how to modify these advanced parameterizations, and (4) what new parameterizations to generate. This description holds for all iterations.

The DockOpt pipeline algorithm can be summarized in a diagram (Figure 1). The docking program DOCK is parameterized by several files, each controlling different aspects of the program’s behavior, such as the sampling algorithm for molecular poses or the scoring function for estimating the free energy of binding for each molecular pose. These files are known as “dockfiles” and exist in custom formats exclusive to DOCK and related software (*e.g.*, matching_spheres.sph). In this work, we use the term *DOCK parameterization* to refer to a specific set of dockfiles, and we take a DOCK parameterization combined with a DOCK executable to constitute a *docking model*.

A DOCK parameterization can be generated from the information contained in a few input files: (1) a receptor structure, (2) a crystallographic ligand structure, and (3) the dockopt_config.yaml parameters file. During the model creation phase (see the arrow labeled “creates” in Figure 1) of the DockOpt pipeline, a directed acyclic graph (*blaster DAG*) is used as the data structure for managing the transformation of the input files (*root nodes*) into several DOCK parameterizations (*leaf nodes*) through a multiplex process involving the controlled variation of numerous intermediate files. An edge in the blaster DAG represents a dependency relation between a certain input–output pair part of a particular step in the pipeline (*i.e.*, a step *s* takes {*x*,···} as input and produces {*y*,···} as output, so output *y* depends on input *x*). For example, matching_spheres.sph depends on rec.crg.pdb in the matching spheres generation step.^51^ A child node can be created only once all of its parent nodes exist. The input files completely determine the blaster DAG, which is automatically derived from them by a deterministic process. Starting from the root nodes of the blaster DAG, a multitude of different branching paths are taken, with each path leading to a distinct leaf node. A specific combination of leaf nodes represents a specific DOCK parameterization, and their respective paths from the root nodes in sum completely define how it is created from the input files. Once initialized, the blaster DAG serves as a pipeline of steps that generates the DOCK parameterizations corresponding to all valid combinations of leaf nodes.

### Parameter Search Algorithms

Two parameter search algorithms are currently supported by DockOpt: grid search and beam search.^52^ Grid search explores the search space through a predefined grid of potential parameter value combinations. Due to its exhaustive nature, this approach can theoretically find the optimal parameter combination(s), given a sufficiently fine discretization of the parameter space. However, when searching through anything but the coarsest resolutions of the parameter space, this method can rapidly become computationally expensive to the point of intractability. In contrast, beam search narrows its search space by applying a selection criterion at each step to retain only the top fraction of candidate DOCK parameterizations for the next step. The range of considered values can be progressively refined, facilitating a more focused exploration of promising solutions. As a result, beam search may not explore all possible options, but it is more computationally efficient as it constrains exploration to regions of the parameter space likely to hold promising parameterizations, based on certain assumptions. For example, one assumption is that locally optimal choices (*i.e.*, high scoring candidate solutions at each step) will yield globally optimal or near-optimal solutions. This may not always be true as locally suboptimal choices can sometimes lead to better overall solutions.

In its prerelease version, DockOpt used grid search as its search algorithm, exhaustively testing all possible combinations of parameter values, with each parameter taking a pool of possible values. For example, distance-to-surface values for electrostatic thin spheres^53^ might be the set {1.0, 1.1, ..., 1.9} and distance-to-surface values for ligand desolvation thin spheres might be the set {0.1, 0.2, ..., 1.0}, resulting in a Cartesian product space of 10 × 10 = 100 combinations. This strategy works well enough for small numbers of parameters, with a limited number of values per parameter. However, it is too inefficient to serve as a general search algorithm as it would subject users to exponentially increasing computational cost when exploring higher-dimensional parameter spaces at finer resolutions. Consequently, the latest release of Pydock3 configures DockOpt to use beam search by default. Below, we demonstrate that our implementation of beam search consistently finds superior parameterizations to those found by grid search using comparable computational expense.

### DockOpt Is Controlled by Parameters in the dockopt_config.yaml File

The dockopt_config.yaml file defines the architecture of the DockOpt pipeline and controls the range of values that is explored for each parameter. The blaster DAG is derived automatically from the settings in the dockopt_config.yaml file, and a single step in a DockOpt pipeline generates several different docking models, which are then evaluated in parallel. A sequence of steps may be defined with optional iteration and/or early stopping, and recursive embedding of step sequences is supported.

### DockOpt Allows Rigorous, Reproducible Experimentation

The DockOpt pipeline comprises predefined step sequences that may be defined once and then reused. Thus, DockOpt greatly simplifies benchmarking by facilitating the rigorous comparison of different DOCK parameterizations, DOCK executables, and even evaluation criteria. Moreover, different evaluation criteria can be applied in different steps within the same DockOpt pipeline, such as using a measure of enrichment first, followed by a measure of pose reproduction, and so on. Therefore, an entire experiment intended to measure the efficacy of several variables or search strategies can be defined in a single DockOpt pipeline and reproduced later simply by rerunning the saved pipeline configuration.

### DockOpt Pipelines Are Flexible

The range of possible pipeline structures in DockOpt is far wider than the default configuration may suggest. Although we recommend that new users try the default configuration first, a wide range of search strategies are available to be explored and customized as users gain more experience and familiarity with the software. These strategies can be tailored to suit the specific requirements of the users’ respective research objectives, providing versatility and flexibility in docking optimization.

### DockOpt Reports

DockOpt generates comprehensive reporting, including a CSV file of results for each docking model tested and an HTML format report containing visualizations, including a histogram of the performance across tested models, also showing a statistical significance threshold; linear–log ROC plots showing enrichment; bar plots for performance of individual multivalued parameters; heatmaps comparing performances across two multivalued parameters; a ridge plot showing the breakdown of energy terms by binary class; and a violin plot showing the charge distribution by binary class.

## Results

New software enabling the automatic optimization of docking models is now available as part of the UCSF DOCK 3.8 release. This software is available for free to academic researchers (see: dock.compbio.ucsf.edu) and at modest cost otherwise (email: dock_industry@googlegroups.com). See the Supporting Information S1 for installation instructions. First, we detail the software’s innovative features, including its automated parameter optimization capabilities. Next, we evaluate the software’s utility by testing it against the 43 targets of the DUDE-Z benchmark.^19^ This evaluation includes a comprehensive statistical analysis to address potential issues of overfitting and to verify the reliability of enrichment metrics, employing methods such as cross-validation and Bonferroni correction. Lastly, we introduce a web service for this software. The resulting docking model can be downloaded and deployed for prospective docking on the user’s on-premises computers, a cloud platform (such as AWS^54^), or any other system capable of large-scale docking.

DockOpt is a single command for generating and evaluating many different docking models when a retrospective data set of molecules is available. The performance of a docking model may be evaluated by retrospective docking, where the ability of the model to distinguish between reported binders (positive class) and presumed nonbinders (negative class) is assessed. DockOpt wraps the generation, evaluation, and optimization of docking models, all in a single tool.

There are dozens of parameters whose values may affect the quality of the docking models produced by DockOpt, but a few tend to have the most impact. For example, the thickness of the low dielectric and ligand desolvation layers are critical in determining the electrostatic and desolvation contributions to the binding energy.^53^ These parameters are essential for accurate scoring of ligand–receptor interactions as they directly influence the prediction of binding affinity. Similarly, the number and placement of matching spheres are paramount in sampling ligand poses.^51^ These “hotspots” are often positioned at energetically favorable sites within the binding pocket and are crucial for guiding the ligand into a bioactive conformation. The optimization of these spheres is thus a key determinant of the docking model’s ability to identify viable binding poses. In this optimization, DockOpt applies a perturbation strategy to the matching spheres, adjusting their placement stochastically. In each step of the pipeline, multiple new matching_spheres.sph files are generated from each existing candidate file, with each new file’s spheres having been perturbed by random samples from a 3D uniform distribution over a ball of a specified radius (*e.g.*, 0.4 Å) centered at the origin. This perturbation radius and the number of new matching_spheres.sph files per candidate are specified in the matching_spheres_perturbation section of dockopt_config.yaml. Moreover, the number of new matching_spheres.sph files per candidate varies across different steps in the pipeline, by default starting at 10 and increasing to 50 in later steps. Further parameters, such as match_goal, bump_maximum, bump_rigid, and chemical_matching, govern the sampling efficiency and the docking accuracy. For instance, match_goal affects the thoroughness of the pose sampling, while bump parameters dictate the stringency of steric clashes between the ligand and the protein. Lastly, chemical_matching determines whether to label and match ligands with receptor sites to improve docking precision by discarding any matches that do not meet specific chemical criteria defined in matching_spheres.sph.^55,56^

DockOpt performs retrospective docking on multiple docking models in parallel using a job scheduler such as Slurm or SGE. Although these two schedulers are the only ones currently supported, it should be straightforward to incorporate any queueing system into DockOpt. After docking, the docking models are evaluated by the specified criterion (*e.g.*, normalized LogAUC) and then ranked by their performance. Depending on the user’s specification of the configuration file controlling the program, the optimization process may repeat until the stopping criterion is met. A report in HTML format of the best parameter set choices is generated, together with figures summarizing all candidate models tested (see the Methods section and below). A CSV file of their performance is saved as results.csv. The parameterizations found to perform best are saved in a dedicated directory best_retrodock_jobs/, along with their predicted poses of all docked molecules in the Tripos Mol2 format. Dockfiles for all other parameterizations can be found in the directory working/, as indexed in results.csv.

In the absence of known ligands, Blastermaster (the successor of blastermaster.py from earlier work^48^) will produce a ready-to-use DOCK parameterizations by means of standard, unoptimized parameter choices.

To evaluate the performance of DockOpt, we benchmarked it against all 43 DUDE-Z targets, using only the data available on the DUDE-Z Web site (dudez2022.docking.org). For instructions on how to obtain DUDE-Z, see the Supporting Information S2.

**Figure 2:**
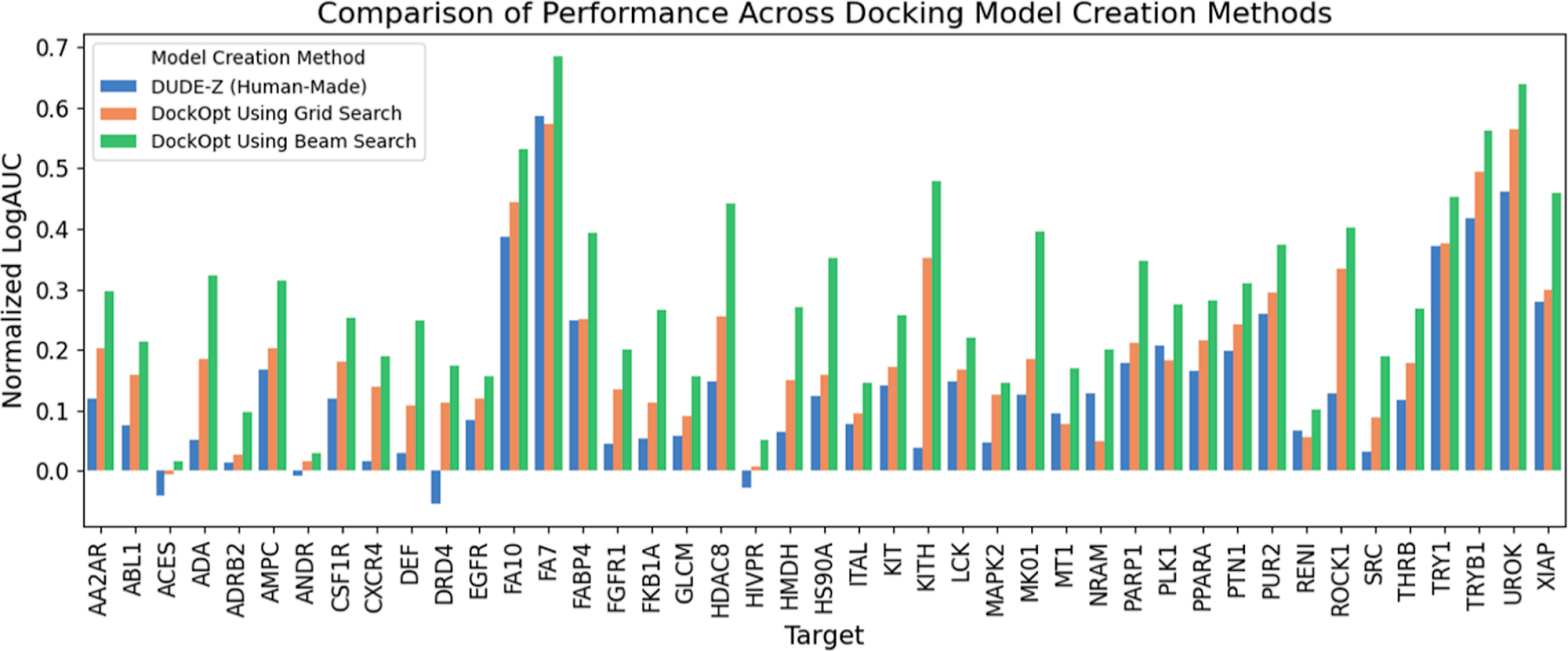
Comparison of beam search and grid search parameter optimizations with the previously published default protocol.^18^

**Figure 3:**
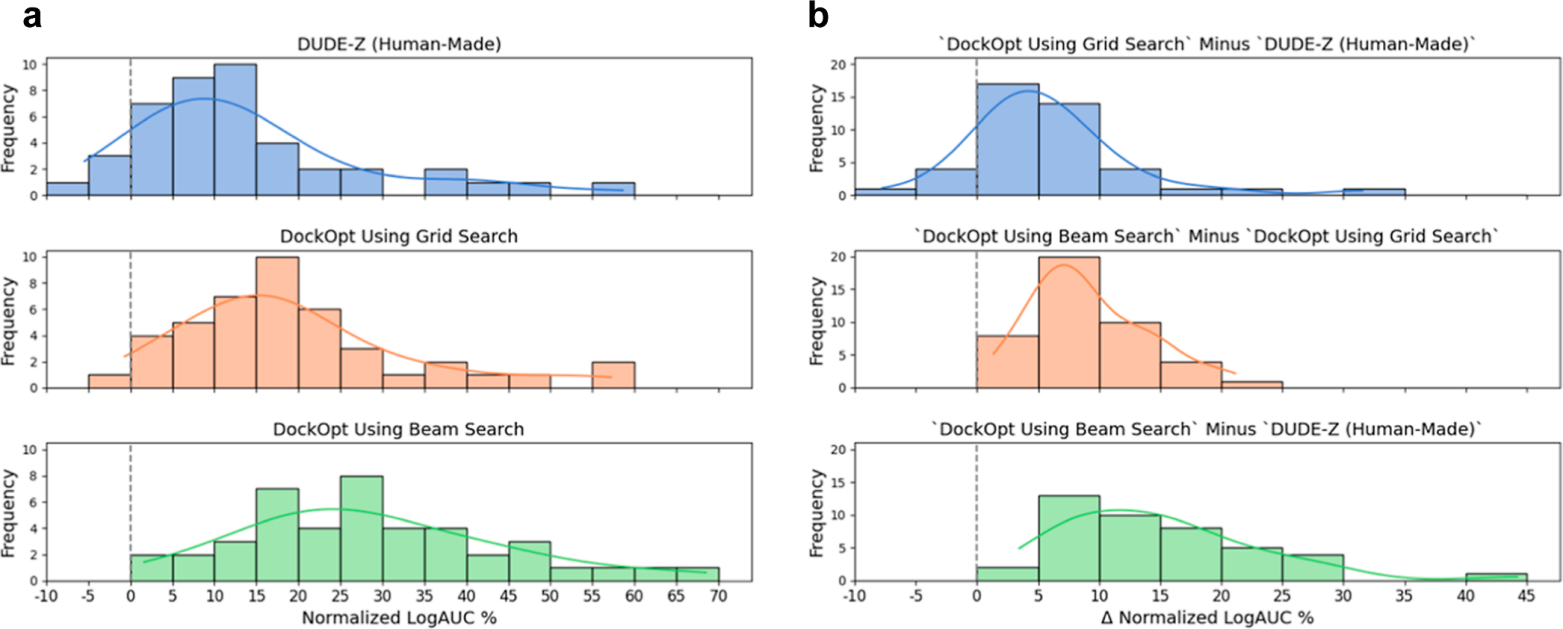
Quality of molecular docking performance as measured by the enrichment of known ligands over property-matched decoys for 43 DUDE-Z systems. Recall that normalized LogAUC satisfies the following: a maximum value of 1 corresponding to a perfect classifier; a value of 0 for a random classifier; a positive value for a better-than-random classifier; and a negative value for a worse-than-random classifier. (3a): Comparison of DOCK parameterizations published in the DUDE-Z paper^19^ (blue) with parameterizations found by the two search algorithms supported by DockOpt, grid search (orange) and beam search (green). (3b): Improvement in enrichment was achieved using DockOpt. Top: from DUDE-Z (human-made) to grid search; middle: from grid search to beam search; and bottom: from DUDE-Z (human-made) to beam search.

For all 43 DUDE-Z targets, the normalized LogAUC (also called the “enrichment score”^50^) of annotated ligands over property-matched decoys produced by the default DockOpt configuration was found to be better than that produced by the human-made DOCK parameterization included for that target in DUDE-Z^19^ (Figure 2), whose parameters are the same as the default parameters of our previous protocol.^19,48^ Comparing the human-made parameterizations published in DUDE-Z to those produced by both grid search and beam search (Figure 3), we observed average increases in normalized LogAUC of 5.7 and 14.9 percentage points, respectively. Using grid search, we observed a maximum increase of 31.6 percentage points for the target KITH, and using beam search, we observed a maximum increase of 44.2 percentage points, also for the target KITH.

**Figure 4:**
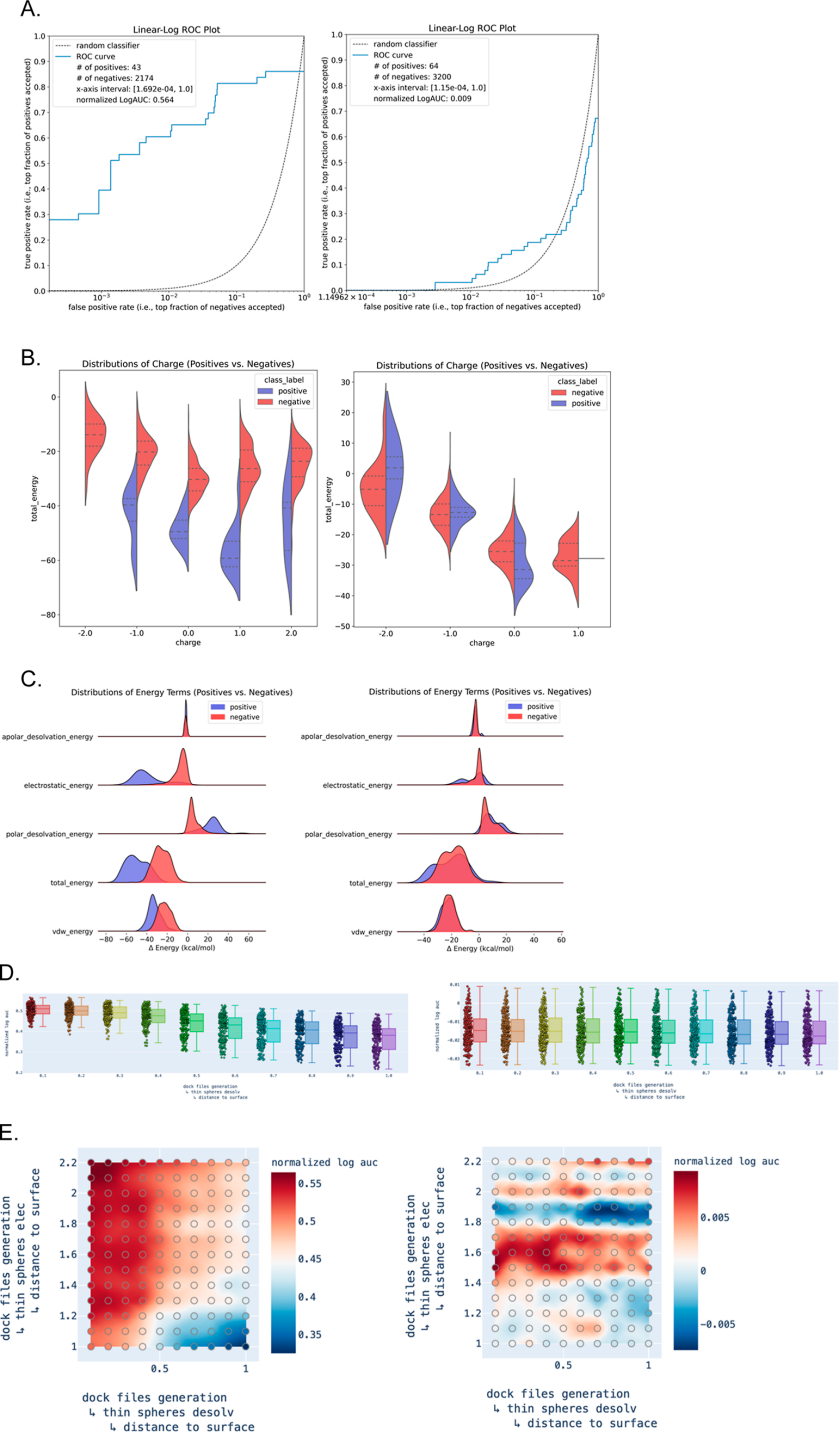
Selected graphical reports of docking models optimized for two targets. Left: coagulation factor VIIa (FA7). Right: HIV-1 protease (HIVPR). (A) Linear–log ROC plot of enrichment of ligands *vs* decoys. (B) Violin plots of the distribution of charges. (C) Unidimensional plots of the distribution of energy terms for docked ligands *vs* decoys. (D) Unidimensional plot of the distributions of enrichment across different values of desolvation thin sphere radii. (E) 2D plots of enrichment as a function of electrostatic and desolvation thin sphere radii.

**Figure 5:**
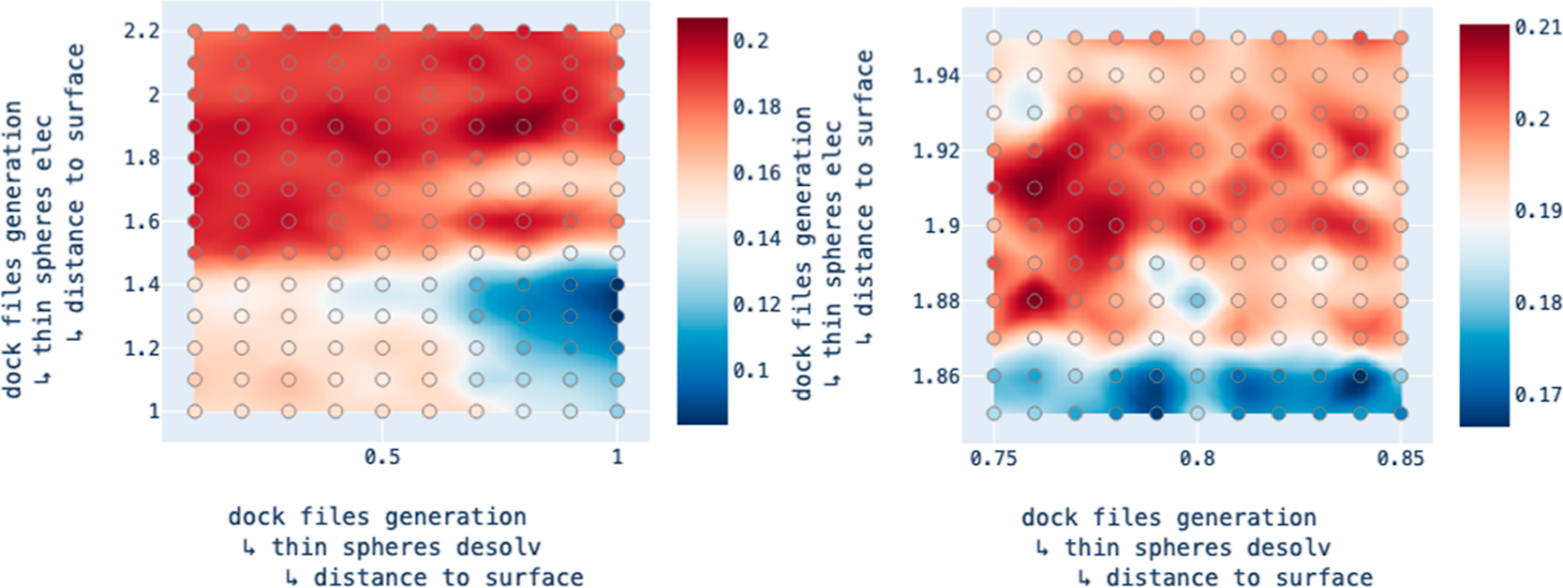
Heatmaps produced by two adjacent steps in a DockOpt pipeline for the target CSF1R, demonstrating the ability of beam search to narrow the range of considered values in a greedy fashion. Left: the heatmap for the former step shows a coarser resolution of exploration with a wider range of parameter values on both axes. The optimum tested coordinate is found to be (0.8, 1.9). Right: the heatmap for the latter step shows a finer resolution of exploration in the neighborhood around the optimum witnessed in the previous step. Note the nontrivial degree of fluctuation in enrichment across tested coordinates, even at a finer resolution.

DockOpt creates a comprehensive report for each target, which can be accessed at dudez2022.docking.org. In this paper, we illustrate the features of these reports using actual results for two DUDE-Z targets as examples, HIV-1 protease (HIVPR) and coagulation factor VIIa (FA7) (Figure 4). Each report includes a linear–log ROC plot of the enrichment of the positive class over the negative class (Figure 4A). This plot captures the performance of a docking model in a single visualization, with the area under the curve (AUC) serving as a quantitative measure of model quality. Next, the report includes additional plots that provide insights into the respective contributions of the terms of the scoring function of the docking program, thereby revealing potential biases that may influence the evaluation of docking models (*e.g.*, imbalanced representations of properties in the data set, such as charge). The split violin charts (Figure 4B) show the scores of the binary classes grouped by net molecular charge. The ridgeline plots (Figure 4C) show how the binary classes compare across the energy terms, whose sum constitutes the predicted free energy of binding. Boxplots of the evaluation criteria are generated for parameters for which multiple values were tested, providing a visual comparison across different parameter values (Figure 4D). Finally, heatmaps (Figure 4E) summarizing the distribution of the evaluation criterion as a function of two variables are generated for every pair of parameters for which multiple values were attempted. The value of each 2D coordinate in the heatmap corresponds to the maximum criterion value obtained across all parameterizations that used the combination of parameter values indicated by the coordinate; for example, a heatmap may show the behavior of the normalized LogAUC as a function of the electrostatic spheres (thin layer) and the desolvation spheres (thin boundary). Returning to the examples of HIVPR and FA7, we observe that HIVPR shows consistently low enrichment across candidate models, irrespective of how the parameters are varied, while FA7 shows a strong dependency on these parameters, thereby indicating the viability of pinpointing an optimal or near-optimal parameter combination for at least some targets. Examining the heatmaps in HTML reports for a pair of consecutive steps exemplifies the ability of the beam search algorithm to effectively “zoom in” on regions of the parameter space that seem more promising (Figure 5). See the Supporting Information S3 for the distribution of the mean best enrichment witnessed across thin sphere radii. Collectively, these autogenerated plots provide the user with a better understanding of the docking models produced by DockOpt, highlight any biases they may have, and help gauge how they would perform in a prospective docking screen. Comprehensive reports featuring these visualizations for all DUDE-Z systems can be accessed at dude2022.docking.org.

**Figure 6:**
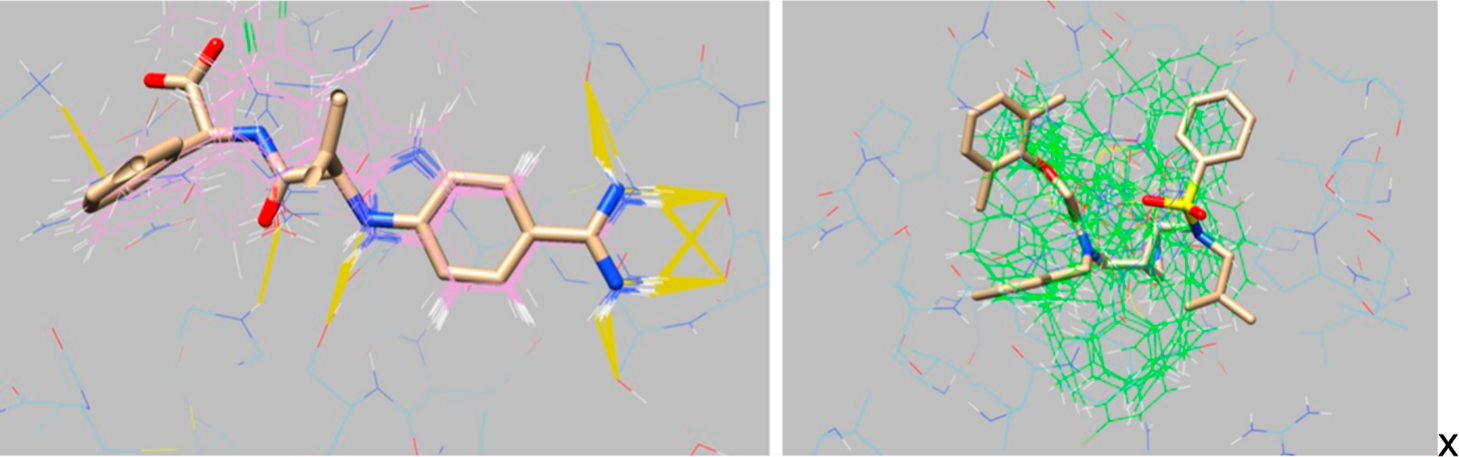
Superimposition of the crystallographic ligand (sticks) and the docked ligands (wire). Hydrogen bonds and polar interactions with the protein are shown in mustard. Left: coagulation factor VIIa (FA7). Right: HIV-1 protease (HIVPR).

Docking models that consistently produce incorrect poses for known ligands are considered flawed, even if they yield a high enrichment in retrospective studies. Although we typically do not know the exact pose of every known ligand in the retrospective data set, we generally expect that most predicted poses should overlap the crystallographic ligand pose and mirror its receptor interactions. We illustrate such pose-oriented considerations in two UCSF Chimera^57^ sessions, each displaying a superimposition of the receptor, the crystallographic ligand, and the predicted poses of the known ligands for comparative analysis (Figure 6). In some cases, a dense superimposition with a significant overlap is observed, typically when a single “warhead” dominates receptor–ligand interactions, as observed with coagulation factor VIIa (FA7). In other cases, the superimposition might be less defined, but it can still confirm that the predicted poses occupy the same region as that of the experimentally observed ligand, as observed with HIV-1 protease (HIVPR). Further comparison with empirical poses of other known HIVPR ligands not included in DUDE-Z reveals that the pose variability observed in this study aligns with the variability observed in said empirical poses (Supporting Information S4).

The normalized LogAUC of a random classifier tends to 0% as the numbers of positive-class molecules and negative-class molecules both go to infinity.^50^ However, whereas a typical retrospective docking campaign usually involves less than 30 known ligands, curated data sets such as DUDE-Z often feature targets with significantly more ligands available, sometimes even reaching the hundreds. To obtain a conditional probability distribution of normalized LogAUC produced by a random classifier given a specific number of positive-class molecules, we performed 1 billion simulations for each number of positives, ranging from 1 to 100, with all data sets maintaining a negative-to-positive ratio of 50:1. The empirical distributions obtained from these simulations (Supporting Information S5) allow us to compute the probability that a perfect random classifier scoring *n* positives and 50*n* negatives would, purely by chance, produce a normalized LogAUC greater than or equal to a given value.

**Figure 7:**
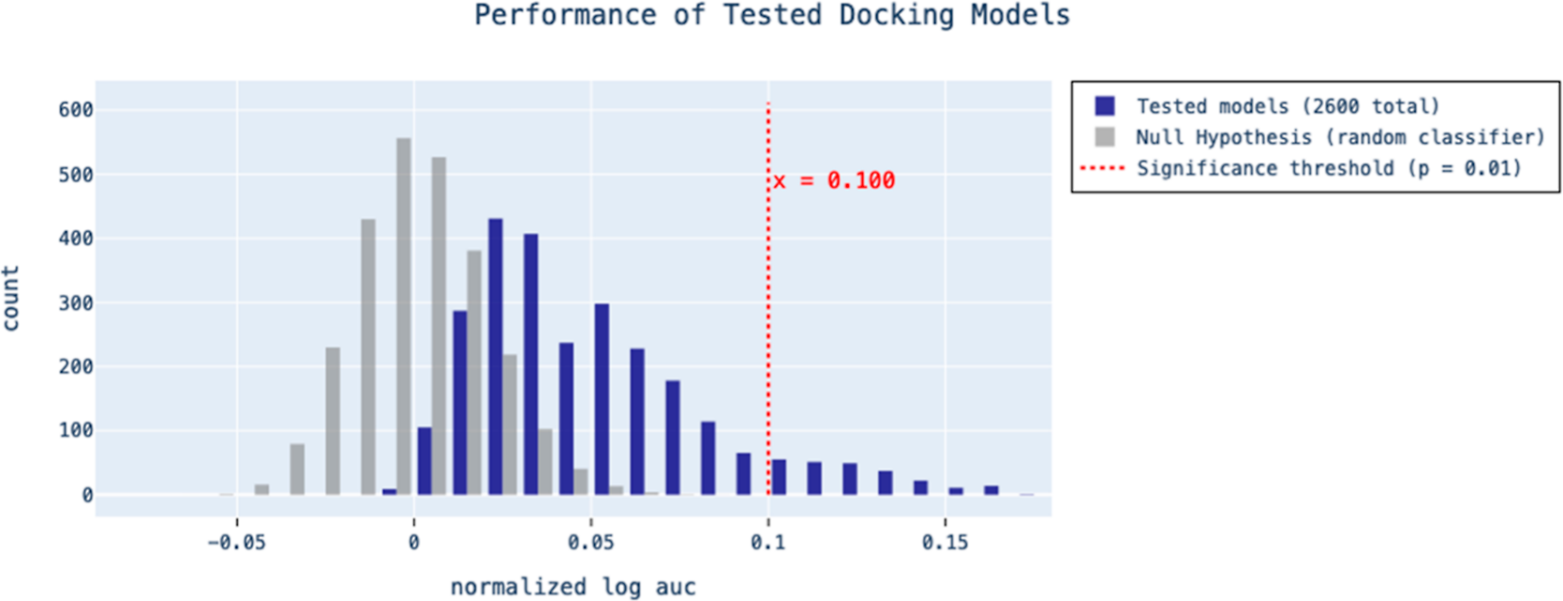
An example histogram of the performance of 2600 tested docking models for target ADA. Using *p* = 0.01 with a Bonferroni correction to account for the multiple tests (2600 total), a significance threshold of 0.100 normalized LogAUC was derived from a cumulative distribution function of the conditional empirical distribution of normalized LogAUC produced by a random classifier. A single tested model’s enrichment exceeding the Bonferroni-corrected significance threshold is all that is necessary to reject the null hypothesis that the enrichment scores observed in the tested models could have come from a random classifier.

To evaluate the possibility that the increased enrichment observed during optimization with DockOpt could simply be due to the large number of docking models tested, we employed the Bonferroni correction to adjust the *p*-value threshold for statistical significance (Figure 7). The Bonferroni correction is a widely used method to control the family wise error rate in multiple hypothesis testing, accounting for the increased likelihood of false positives when conducting a larger number of tests. The correction involves dividing the desired *p*-value by the number of tests performed; for example, a *p*-value of *p* = 0.01 (the default in DockOpt) corrected for 1000 tests would become 10^–5^. The Bonferroni correction assumes that all tests performed are independent, which is not necessarily the case for parameterizations generated by DockOpt, as parameterizations nearby in the parameter space are expected to yield similar results. However, the significance threshold obtained by the Bonferroni correction is strictly more stringent than that obtained by any method that accounts for dependent tests; therefore, using it only further mitigates the risk of falsely identifying insignificant results as significant. Consequently, it is important to recognize that this conservative approach may increase the risk of false negatives, particularly in contexts of high test correlation. However, the priority generally lies in avoiding false positives.

By implementing the Bonferroni correction in our analysis, we can more rigorously evaluate the statistical significance of the observed enrichments. This safeguards against attributing apparent improvements in docking performance to the mere artifact of having tested multiple docking models. Our findings substantiate that the performance superiority of DockOpt over the previously published DUDE-Z parameterizations remains statistically significant even after adjusting for multiple comparisons, thereby offering a reliable metric for assessing the efficacy of our method.

**Figure 8:**
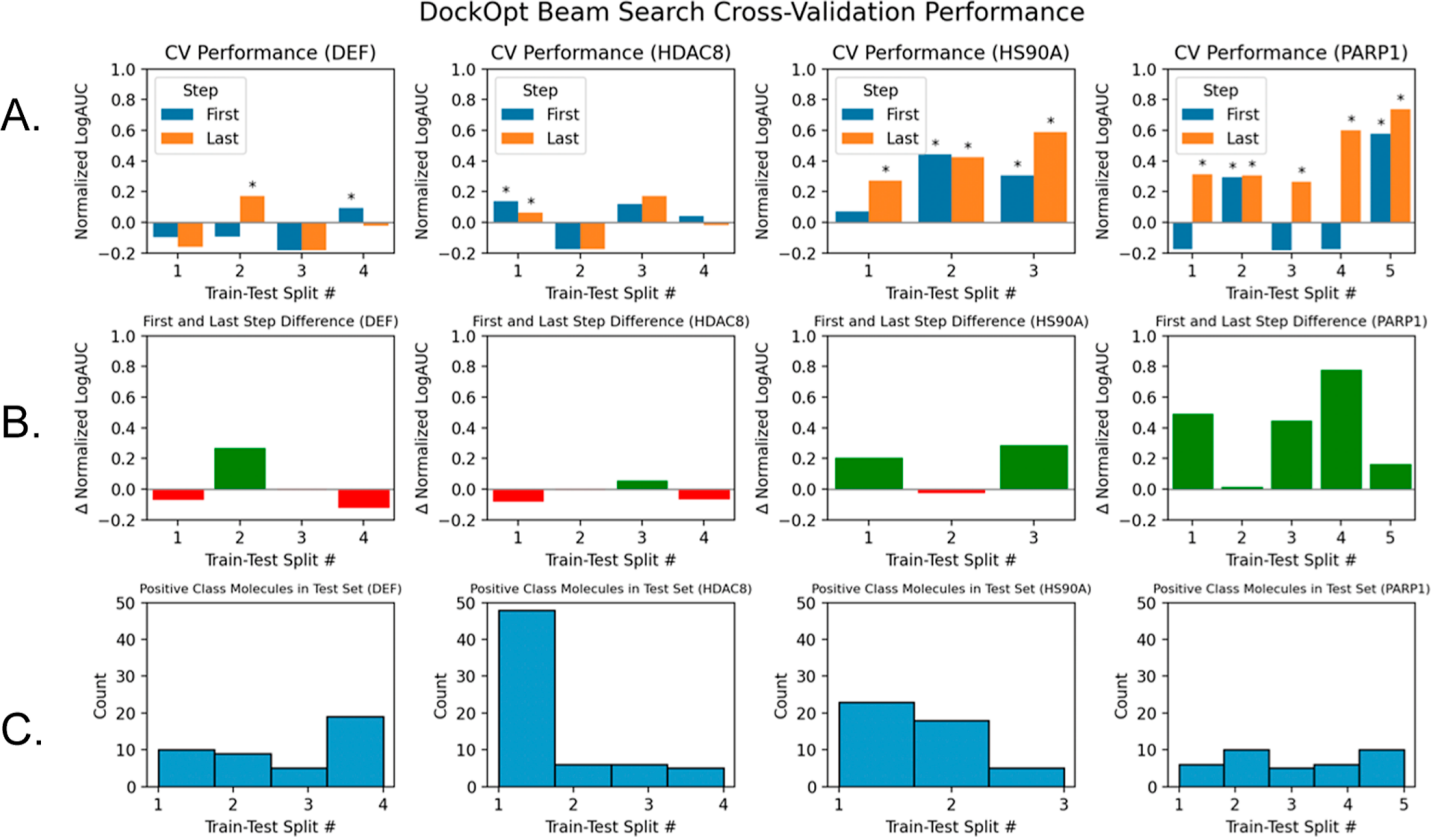
Cross-validation performance of four DUDE-Z targets with a substantial improvement in enrichment exhibited when transitioning from the grid search to beam search. (A) Grouped bar plot comparison of test set enrichment between DockOpt beam search models produced in the first step and the last step. Black asterisks are placed above each bar for which the normalized LogAUC surpasses the threshold for statistical significance that the corresponding docking model is superior to a random classifier (*p* = 0.01). Note that this calculation of significance also depends on the number of positive class molecules (row C). (B) Bar plots showing the difference in performance when transitioning from the first step of beam search to the last step. Red bars indicate worse performance, while green bars indicate improved performance. (C) Number of positive class molecules in each test set of the cross-validation data sets.

Despite the DOCK scoring function being grounded in physics, beam search theoretically has the potential to overfit a docking model to the retrospective data set being used for optimization. To test for evidence of overfitting, we selected four targets that demonstrated a significant increase in enrichment when transitioning from grid search to the beam search. This substantial improvement in performance made these targets prime candidates for potential overfitting. For each target, we clustered their positive class molecules by Morgan fingerprint Tanimoto coefficient (Tc) similarity such that each cluster contained only those molecules with a maximum Tc distance of 0.6 away from each other. Next, we employed a leave-one-out cross-validation strategy to create multiple data sets for each target, each comprising a training set and a test set. We then ran beam search on each training set and performed retrospective docking on the corresponding test sets for each docking model produced at the end of the beam search steps (Figure 8). It should be noted that completion of the first step of beam search is functionally equivalent to performing the retrospective controls advised in the published lab protocol.^18^ Therefore, comparing the cross-validation performance of the docking models produced by the first and last steps of beam search allows us to check whether the iteration of beam search steps causes worsening performance with respect to test set enrichment. Upon comparison, we find no evidence for worse cross-validation performance in the final models produced by the beam search (Figure 8B). Moreover, the overall trends observed in cross-validation performance throughout the beam search steps do not suggest overfitting, as evidenced by the lack of clear improvement across targets when employing early stopping (Supporting Information S6). Our cross-validation analysis confirms that DockOpt can optimize docking models effectively, with no evidence of overfitting observed across the various steps of the beam search process.

**Figure 9:**
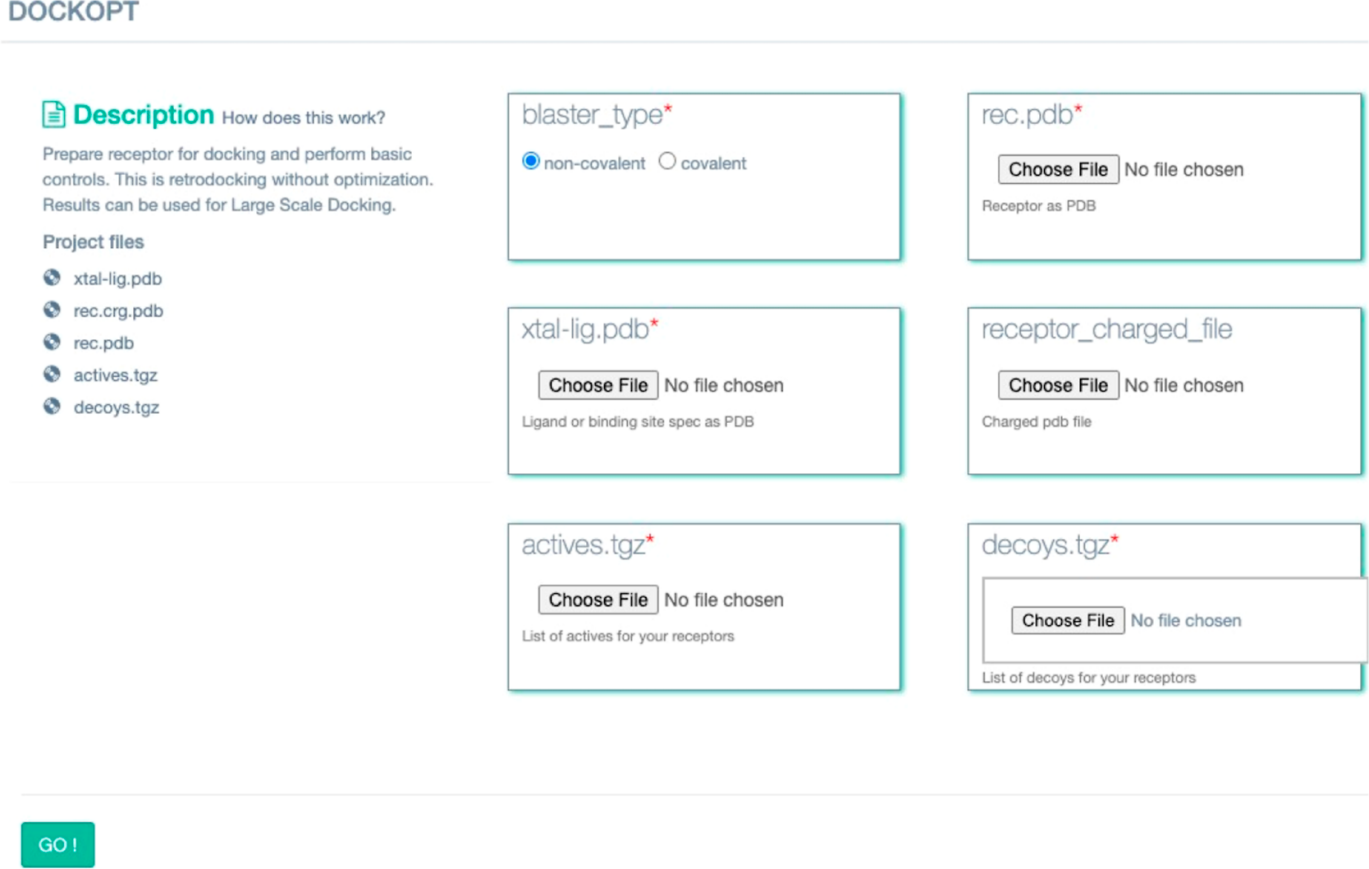
Web Interface for DockOpt *via*tldr.docking.org.

Encouraged by the ability of DockOpt to produce docking models apparently suitable for prospective docking (Figure 3), we built a web-based interface for it at tldr.docking.org under the “DockOpt” module (Figure 9). Registration is free. Sample data for 43 targets in ready-to-use formats are available at dudez2022.docking.org.

This regression was calculated with the constraint that the y-intercept be equal to 2 h, corresponding to the minimum time necessary to generate the candidate docking models using the default DockOpt configuration.

**Figure 10:**
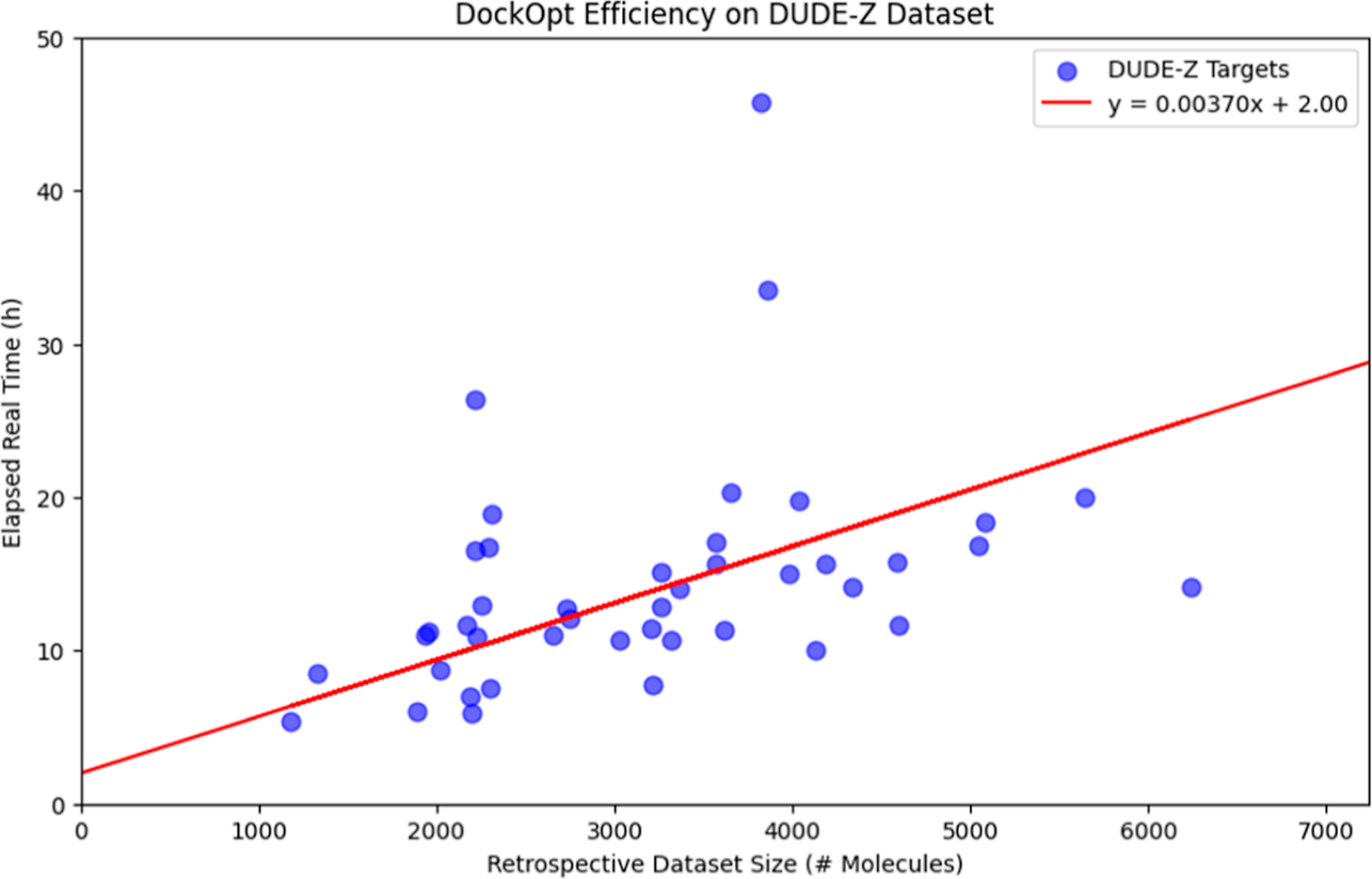
Linear regression on DockOpt runtime *vs* retrospective data set size. The variability in observed runtimes from the regression model can be primarily attributed to the varying levels of exploration required by the parameter search algorithm, depending on the characteristics of the target data and the retrospective data set.

DockOpt jobs using the default configuration can be expected to take up to a day for larger retrospective data sets of several thousands of molecules (Figure 10), though the exact time will depend on the server load as well as to what degree the parameter search algorithm converges to similar parameterizations over time. The resultant model can be deployed in a large-scale docking screen on any system with the necessary compute resources, such as a departmental cluster or *via* a cloud platform (*e.g.*, AWS^54^).

## Discussion

Three themes emerge from this study. **First**, a new automated pipeline for docking model creation, evaluation, and optimization has been developed. **Second**, the automated procedure can optimize the docking parameters significantly better than our previous automated system and, for most targets, produces docking models that are suitable for large-scale prospective screens. **Third**, the new pipeline can be installed locally or accessed *via* a new web interface we have created, and the resultant docking model may be downloaded and deployed for large-scale docking, either on premises or in the cloud.^54^ We take up each of these themes in turn.

DockOpt implements many of the best practices in our standard lab protocol^18^ in an automated fashion. We have augmented this procedure to include optimization techniques that capture many, but not all, of the current best practices in our lab. For example, the boundaries of the regions of low dielectric and ligand desolvation in the binding site are optimized. Work in our lab suggests that defining these boundaries is often critical to obtaining satisfactory retrospective enrichment during model optimization.^53^ The pipeline also optimizes the matching spheres used for sampling ligand orientations, which play a key role in determining whether a docking model is likely to succeed in a prospective screen. Furthermore, the software has been designed with flexibility in mind, allowing for the optimization of dozens of additional parameters, and minimal development effort is required to incorporate and optimize any new parameters or search strategies that a researcher might consider desirable.

DockOpt demonstrates competent docking performance against the DUDE-Z benchmark regularly used in our research. In 36 out of the 43 DUDE-Z systems (∼84%), this fully automated procedure with a beam search produces a docking model exhibiting a normalized LogAUC of at least 15%. This threshold is a heuristic for whether a docking model is suitable for prospective docking. This performance represents a striking improvement compared to the human-made docking models published in DUDE-Z, which resulted in only 13 successes (∼30%) with the same standard. While the current use of enrichment metrics for model evaluation by DockOpt enables the direct comparison of performance between DockOpt and past methodologies that also rely on enrichment metrics, we acknowledge that the inclusion of pose reproduction metrics is essential for a comprehensive evaluation of docking model quality. The modular design of DockOpt supports the future integration of such metrics, making it suitable for use in the earliest stages of drug discovery, including targets for which known hits are not yet available.

The use of the Bonferroni correction and cross-validation strategy in our analysis provides valuable checks for judging the efficacy of DockOpt. By adjustment of the significance threshold to account for the use of multiple tests, the Bonferroni correction effectively reduces the probability of Type I errors (*i.e.*, false positives). This statistical correction serves as a key control mechanism, providing confidence that the performance improvements we report in this work are not merely statistical contrivances but indeed genuine indicators of the ability of DockOpt to effectively optimize docking models. Although the Bonferroni correction assumes that the tests are independent, which is likely not the case with DockOpt, it nevertheless provides a reliable conservative measure of statistical significance. Additionally, we bolstered our evaluation of docking model quality using a cross-validation strategy, allowing us to check for overfitting. The results from this cross-validation show no evidence of overfitting and in fact imply an improvement in test performance beyond that exhibited by models created using our standard lab protocol,^18^ suggesting the ability of DockOpt to effectively optimize docking models. This combined approach of using the Bonferroni correction and cross-validation significantly increases our confidence in the robustness and reliability of DockOpt.

We set up a public web interface for DockOpt at https://tldr.docking.org, which showcases this software’s ability to build and refine docking models completely automatically, given a retrospective data set of molecules. This platform also evaluates the resultant model’s suitability for prospective docking screens, estimating the likelihood of the model to consistently prioritize new ligands. Additional tools that automate other aspects of the docking process, including decoy generation, are also available at https://tldr.docking.org.

There are several caveats to this work. The latest version of DockOpt still requires a retrospective data set to perform optimization. Without these molecules serving as controls, DockOpt cannot assess whether a given model is of sufficient quality to warrant its use for prospective docking. While its current reliance on known ligands initially seems to limit DockOpt’s utility to hit expansion rather than hit discovery, the software is structured to support the incorporation of new evaluation criteria, including ligand-free ones. Until these new criteria are supported, in the absence of known ligands, we recommend using Blastermaster, which employs standard default values for the parameters otherwise optimized by DockOpt. Moreover, because measures of enrichment capacity do not incorporate any structural information on predicted poses, the use of normalized LogAUC as the single criterion for model evaluation also means that there is the possibility of the model overfitting to the ligands provided by the user, which usually number at most a few dozen. However, this potential issue could be ameliorated by using an alternate criterion measuring pose reproduction or perhaps even a criterion measuring both enrichment and pose reproduction. Forthcoming updates to DockOpt are expected to enhance the tool’s capability to evaluate the geometric accuracy of the ligand poses it generates, ensuring that the predicted interactions and binding conformations agree with known structural data. Lastly, it must be noted that while DockOpt is effective on most DUDE-Z targets, it does not work on all of them. Therefore, our approach should not be mistaken as a universal solution to automatic optimization of docking models.

These caveats should not obscure the main results of this work. We developed a fully automated tool for the creation, evaluation, and optimization of docking models, which is now available as part of the UCSF DOCK 3.8 distribution. In addition to being offered free of charge to academic researchers *via*https://dock.compbio.ucsf.edu, the software can also be used for free by nonacademic researchers upon registration at https://tldr.docking.org. It is important to note that we cannot guarantee the results of any docking screen using DockOpt; this software is to be utilized at the user’s own risk. For the best outcomes, we strongly encourage the use of sanity checks and controls at every stage of the docking process, as discussed throughout this work.

## Data Availability

The software DockOpt and Blastermaster are part of DOCK 3.8, which is distributed using a UCSF license. This software is free to academics (dock.compbio.ucsf.edu for a free license and download) and at modest cost to for-profit users. (dock_industry@googlegroups.com). All data, including the DUDE-Z benchmark, and all results reported in this paper are available from dudez2022.docking.org. These data are available via a CC BY 4.0 license.

## Supporting Information Available

Documentation about Pydock3, how to install it, and how to use its tools, including Dock Opt and Blastermaster; documentation about the DOCK-Z data set toolkit, used to benchmark DockOpt against DUDE-Z; empirical distributions of best normalized LogAUC for thin sphere radii parameters across DUDE-Z targets; empiriacal poses for known ligands for HIVPR not included in DUDE-Z; empirical distributions approximating the behavior of a truly known random classifier in terms of normalized LogAUC; and cross-validation performance over all steps of beam searh of four DUDE-Z targets that exhibited a significant improvement when transitioning from grid search to beam search (PDF)

## References

[ref1] Grebner C.; Malmerberg E.; Shewmaker A.; Batista J.; Nicholls A.; Sadowski J. Virtual Screening in the Cloud: How Big Is Big Enough?. J. Chem. Inf. Model. 2020, 60, 4274–4282. 10.1021/acs.jcim.9b00779. 31682421

[ref2] Zhu H.; Zhang Y.; Li W.; Huang N. A Comprehensive Survey of Prospective Structure-Based Virtual Screening for Early Drug Discovery in the Past Fifteen Years. Int. J. Mol. Sci. 2022, 23, 15961 10.3390/ijms232415961. 36555602 PMC9781938

[ref3] Muegge I.; Bergner A.; Kriegl J. M. Computer-Aided Drug Design at Boehringer Ingelheim. J. Comput.-Aided Mol. Des. 2017, 31, 275–285. 10.1007/s10822-016-9975-3. 27650777

[ref4] Lyu J.; Wang S.; Balius T. E.; Singh I.; Levit A.; Moroz Y. S.; O’Meara M. J.; Che T.; Algaa E.; Tolmachova K.; Tolmachev A. A.; Shoichet B. K.; Roth B. L.; Irwin J. J. Ultra-Large Library Docking for Discovering New Chemotypes. Nature 2019, 566, 224–229. 10.1038/s41586-019-0917-9. 30728502 PMC6383769

[ref5] Stein R. M.; Kang H. J.; McCorvy J. D.; Glatfelter G. C.; Jones A. J.; Che T.; Slocum S.; Huang X. P.; Savych O.; Moroz Y. S.; Stauch B.; Johansson L. C.; Cherezov V.; Kenakin T.; Irwin J. J.; Shoichet B. K.; Roth B. L.; Dubocovich M. L. Virtual Discovery of Melatonin Receptor Ligands to Modulate Circadian Rhythms. Nature 2020, 579, 609–614. 10.1038/s41586-020-2027-0. 32040955 PMC7134359

[ref6] Gorgulla C.; Boeszoermenyi A.; Wang Z. F.; Fischer P. D.; Coote P. W.; Padmanabha Das K. M.; Malets Y. S.; Radchenko D. S.; Moroz Y. S.; Scott D. A.; Fackeldey K.; Hoffmann M.; Iavniuk I.; Wagner G.; Arthanari H. An Open-Source Drug Discovery Platform Enables Ultra-Large Virtual Screens. Nature 2020, 580, 663–668. 10.1038/s41586-020-2117-z. 32152607 PMC8352709

[ref7] Sadybekov A. A.; Brouillette R. L.; Marin E.; Sadybekov A. V.; Luginina A.; Gusach A.; Mishin A.; Besserer-Offroy E.; Longpre J. M.; Borshchevskiy V.; Cherezov V.; Sarret P.; Katritch V. Structure-Based Virtual Screening of Ultra-Large Library Yields Potent Antagonists for a Lipid Gpcr. Biomolecules 2020, 10, 1634 10.3390/biom10121634. 33287369 PMC7761830

[ref8] Alon A.; Lyu J.; Braz J. M.; Tummino T. A.; Craik V.; O’Meara M. J.; Webb C. M.; Radchenko D. S.; Moroz Y. S.; Huang X. P.; Liu Y.; Roth B. L.; Irwin J. J.; Basbaum A. I.; Shoichet B. K.; Kruse A. C. Structures of the σ2 receptor enable docking for bioactive ligand discovery. Nature 2021, 600, 759–764. 10.1038/s41586-021-04175-x. 34880501 PMC8867396

[ref9] Kaplan A. L.; Confair D. N.; Kim K.; Barros-Alvarez X.; Rodriguiz R. M.; Yang Y.; Kweon O. S.; Che T.; McCorvy J. D.; Kamber D. N.; Phelan J. P.; Martins L. C.; Pogorelov V. M.; DiBerto J. F.; Slocum S. T.; Huang X. P.; Kumar J. M.; Robertson M. J.; Panova O.; Seven A. B.; Wetsel A. Q.; Wetsel W. C.; Irwin J. J.; Skiniotis G.; Shoichet B. K.; Roth B. L.; Ellman J. A. Bespoke Library Docking for 5-Ht(2a) Receptor Agonists with Antidepressant Activity. Nature 2022, 610, 582–591. 10.1038/s41586-022-05258-z. 36171289 PMC9996387

[ref10] Fink E. A.; Xu J.; Hubner H.; Braz J. M.; Seemann P.; Avet C.; Craik V.; Weikert D.; Schmidt M. F.; Webb C. M.; Tolmachova N. A.; Moroz Y. S.; Huang X. P.; Kalyanaraman C.; Gahbauer S.; Chen G.; Liu Z.; Jacobson M. P.; Irwin J. J.; Bouvier M.; Du Y.; Shoichet B. K.; Basbaum A. I.; Gmeiner P. Structure-based discovery of nonopioid analgesics acting through the α _2A_ -adrenergic receptor. Science 2022, 377, eabn7065 10.1126/science.abn7065. 36173843 PMC10360211

[ref11] Gahbauer S.; Correy G. J.; Schuller M.; Ferla M. P.; Doruk Y. U.; Rachman M.; Wu T.; Diolaiti M.; Wang S.; Neitz R. J.; Fearon D.; Radchenko D. S.; Moroz Y. S.; Irwin J. J.; Renslo A. R.; Taylor J. C.; Gestwicki J. E.; von Delft F.; Ashworth A.; Ahel I.; Shoichet B. K.; Fraser J. S. Iterative Computational Design and Crystallographic Screening Identifies Potent Inhibitors Targeting the Nsp3Macrodomain of Sars-Cov-2. Proc. Natl. Acad. Sci. U.S.A. 2023, 120, e2212931120 10.1073/pnas.2212931120. 36598939 PMC9926234

[ref12] Singh I.; Li F.; Fink E. A.; Chau I.; Li A.; Rodriguez-Hernandez A.; Glenn I.; Zapatero-Belinchon F. J.; Rodriguez M. L.; Devkota K.; Deng Z.; White K.; Wan X.; Tolmachova N. A.; Moroz Y. S.; Kaniskan H. U.; Ott M.; Garcia-Sastre A.; Jin J.; Fujimori D. G.; Irwin J. J.; Vedadi M.; Shoichet B. K. Structure-Based Discovery of Inhibitors of the Sars-Cov-2 Nsp14 N7-Methyltransferase. J. Med. Chem. 2023, 66, 7785–7803. 10.1021/acs.jmedchem.2c02120. 37294077 PMC10374283

[ref13] Sadybekov A. A.; Sadybekov A. V.; Liu Y.; Iliopoulos-Tsoutsouvas C.; Huang X. P.; Pickett J.; Houser B.; Patel N.; Tran N. K.; Tong F.; Zvonok N.; Jain M. K.; Savych O.; Radchenko D. S.; Nikas S. P.; Petasis N. A.; Moroz Y. S.; Roth B. L.; Makriyannis A.; Katritch V. Synthon-Based Ligand Discovery in Virtual Libraries of over 11 Billion Compounds. Nature 2022, 601, 452–459. 10.1038/s41586-021-04220-9. 34912117 PMC9763054

[ref14] Jumper J.; Evans R.; Pritzel A.; Green T.; Figurnov M.; Ronneberger O.; Tunyasuvunakool K.; Bates R.; Zidek A.; Potapenko A.; Bridgland A.; Meyer C.; Kohl S. A. A.; Ballard A. J.; Cowie A.; Romera-Paredes B.; Nikolov S.; Jain R.; Adler J.; Back T.; Petersen S.; Reiman D.; Clancy E.; Zielinski M.; Steinegger M.; Pacholska M.; Berghammer T.; Bodenstein S.; Silver D.; Vinyals O.; Senior A. W.; Kavukcuoglu K.; Kohli P.; Hassabis D. Highly Accurate Protein Structure Prediction with Alphafold. Nature 2021, 596, 583–589. 10.1038/s41586-021-03819-2. 34265844 PMC8371605

[ref15] Webb B.; Sali A. Comparative Protein Structure Modeling Using Modeller. Curr. Protoc. Bioinf. 2016, 86, 2.9.1 10.1002/cpps.20. 27801516

[ref16] Baek M.; DiMaio F.; Anishchenko I.; Dauparas J.; Ovchinnikov S.; Lee G. R.; Wang J.; Cong Q.; Kinch L. N.; Schaeffer R. D.; Millan C.; Park H.; Adams C.; Glassman C. R.; DeGiovanni A.; Pereira J. H.; Rodrigues A. V.; van Dijk A. A.; Ebrecht A. C.; Opperman D. J.; Sagmeister T.; Buhlheller C.; Pavkov-Keller T.; Rathinaswamy M. K.; Dalwadi U.; Yip C. K.; Burke J. E.; Garcia K. C.; Grishin N. V.; Adams P. D.; Read R. J.; Baker D. Accurate Prediction of Protein Structures and Interactions Using a Three-Track Neural Network. Science 2021, 373, 871–876. 10.1126/science.abj8754. 34282049 PMC7612213

[ref17] Altschul S. F.; Gish W.; Miller W.; Myers E. W.; Lipman D. J. Basic Local Alignment Search Tool. J. Mol. Biol. 1990, 215, 403–410. 10.1016/S0022-2836(05)80360-2. 2231712

[ref18] Bender B. J.; Gahbauer S.; Luttens A.; Lyu J.; Webb C. M.; Stein R. M.; Fink E. A.; Balius T. E.; Carlsson J.; Irwin J. J.; Shoichet B. K. A Practical Guide to Large-Scale Docking. Nat. Protoc. 2021, 16, 4799–4832. 10.1038/s41596-021-00597-z. 34561691 PMC8522653

[ref19] Stein R. M.; Yang Y.; Balius T. E.; O’Meara M. J.; Lyu J.; Young J.; Tang K.; Shoichet B. K.; Irwin J. J. Property-Unmatched Decoys in Docking Benchmarks. J. Chem. Inf. Model. 2021, 61, 699–714. 10.1021/acs.jcim.0c00598. 33494610 PMC7913603

[ref20] Irwin J. J.; Shoichet B. K.; Mysinger M. M.; Huang N.; Colizzi F.; Wassam P.; Cao Y. Automated Docking Screens: A Feasibility Study. J. Med. Chem. 2009, 52, 5712–5720. 10.1021/jm9006966. 19719084 PMC2745826

[ref21] Gorgulla C.; Padmanabha Das K. M.; Leigh K. E.; Cespugli M.; Fischer P. D.; Wang Z. F.; Tesseyre G.; Pandita S.; Shnapir A.; Calderaio A.; Gechev M.; Rose A.; Lewis N.; Hutcheson C.; Yaffe E.; Luxenburg R.; Herce H. D.; Durmaz V.; Halazonetis T. D.; Fackeldey K.; Patten J. J.; Chuprina A.; Dziuba I.; Plekhova A.; Moroz Y.; Radchenko D.; Tarkhanova O.; Yavnyuk I.; Gruber C.; Yust R.; Payne D.; Naar A. M.; Namchuk M. N.; Davey R. A.; Wagner G.; Kinney J.; Arthanari H. A Multi-Pronged Approach Targeting Sars-Cov-2 Proteins Using Ultra-Large Virtual Screening. iScience 2021, 24, 102021 10.1016/j.isci.2020.102021. 33426509 PMC7783459

[ref22] Arul Murugan N.; Ruba Priya G.; Narahari Sastry G.; Markidis S. Artificial Intelligence in Virtual Screening: Models Versus Experiments. Drug Discovery Today 2022, 27, 1913–1923. 10.1016/j.drudis.2022.05.013. 35597513

[ref23] Murail S.; de Vries S. J.; Rey J.; Moroy G.; Tuffery P. Seamdock: An Interactive and Collaborative Online Docking Resource to Assist Small Compound Molecular Docking. Front Mol. Biosci 2021, 8, 716466 10.3389/fmolb.2021.716466. 34604303 PMC8484321

[ref24] Kochnev Y.; Hellemann E.; Cassidy K. C.; Durrant J. D. Webina: An Open-Source Library and Web App That Runs Autodock Vina Entirely in the Web Browser. Bioinformatics 2020, 36, 4513–4515. 10.1093/bioinformatics/btaa579. 32559277 PMC7575045

[ref25] Grosdidier A.; Zoete V.; Michielin O. Swissdock, a Protein-Small Molecule Docking Web Service Based on Eadock Dss. Nucleic Acids Res. 2011, 39, W270–W277. 10.1093/nar/gkr366. 21624888 PMC3125772

[ref26] Guedes I. A.; Costa L. S. C.; Dos Santos K. B.; Karl A. L. M.; Rocha G. K.; Teixeira I. M.; Galheigo M. M.; Medeiros V.; Krempser E.; Custodio F. L.; Barbosa H. J. C.; Nicolas M. F.; Dardenne L. E. Drug Design and Repurposing with Dockthor-Vs Web Server Focusing on Sars-Cov-2 Therapeutic Targets and Their Non-Synonym Variants. Sci. Rep. 2021, 11, 5543 10.1038/s41598-021-84700-0. 33692377 PMC7946942

[ref27] Wang J.; Dokholyan N. V. Medusadock 2.0: Efficient and Accurate Protein-Ligand Docking with Constraints. J. Chem. Inf. Model. 2019, 59, 2509–2515. 10.1021/acs.jcim.8b00905. 30946779 PMC6597311

[ref28] McNutt A. T.; Francoeur P.; Aggarwal R.; Masuda T.; Meli R.; Ragoza M.; Sunseri J.; Koes D. R. Gnina 1.0: Molecular Docking with Deep Learning. J. Cheminf. 2021, 13, 43 10.1186/s13321-021-00522-2. PMC8191141 34108002

[ref29] Li H.; Sze K.-H.; Lu G.; Ballester P. J. Machine-Learning Scoring Functions for Structure-Based Drug Lead Optimization. Wires Computational Molecular Science 2020, 10, e1465 10.1002/wcms.1465.

[ref30] Poli G.; Martinelli A.; Tuccinardi T. Reliability Analysis and Optimization of the Consensus Docking Approach for the Development of Virtual Screening Studies. J. Enzyme Inhib. Med. Chem. 2016, 31, 167–173. 10.1080/14756366.2016.1193736. 27311630

[ref31] Ng M. C.; Fong S.; Siu S. W. Psovina: The Hybrid Particle Swarm Optimization Algorithm for Protein-Ligand Docking. J. Bioinform Comput. Biol. 2015, 13, 1541007 10.1142/S0219720015410073. 25800162

[ref32] Alekseenko A.; Kotelnikov S.; Ignatov M.; Egbert M.; Kholodov Y.; Vajda S.; Kozakov D. Cluspro Ligtbm: Automated Template-Based Small Molecule Docking. J. Mol. Biol. 2020, 432, 3404–3410. 10.1016/j.jmb.2019.12.011. 31863748 PMC7890944

[ref33] Labbe C. M.; Rey J.; Lagorce D.; Vavrusa M.; Becot J.; Sperandio O.; Villoutreix B. O.; Tuffery P.; Miteva M. A. Mtiopenscreen: A Web Server for Structure-Based Virtual Screening. Nucleic Acids Res. 2015, 43, W448–W454. 10.1093/nar/gkv306. 25855812 PMC4489289

[ref34] Li H.; Leung K. S.; Ballester P. J.; Wong M. H. Istar: A Web Platform for Large-Scale Protein-Ligand Docking. PLoS One 2014, 9, e85678 10.1371/journal.pone.0085678. 24475049 PMC3901662

[ref35] Pires D. E. V.; Veloso W. N. P.; Myung Y.; Rodrigues C. H. M.; Silk M.; Rezende P. M.; Silva F.; Xavier J. S.; Velloso J. P. L.; da Silveira C. H.; Ascher D. B. Easyvs: A User-Friendly Web-Based Tool for Molecule Library Selection and Structure-Based Virtual Screening. Bioinformatics 2020, 36, 4200–4202. 10.1093/bioinformatics/btaa480. 32399551

[ref36] Tsai T. Y.; Chang K. W.; Chen C. Y. Iscreen: World’s First Cloud-Computing Web Server for Virtual Screening and De Novo Drug Design Based on Tcm Database@Taiwan. J. Comput.-Aided Mol. Des. 2011, 25, 525–531. 10.1007/s10822-011-9438-9. 21647737

[ref37] Gorgulla C.; Cinaroglu S. S.; Fischer P. D.; Fackeldey K.; Wagner G.; Arthanari H. Virtualflow Ants-Ultra-Large Virtual Screenings with Artificial Intelligence Driven Docking Algorithm Based on Ant Colony Optimization. Int. J. Mol. Sci. 2021, 22, 5807 10.3390/ijms22115807. 34071676 PMC8199267

[ref38] Zhang B.; Li H.; Yu K.; Jin Z. Molecular Docking-Based Computational Platform for High-Throughput Virtual Screening. CCF Trans High Perform Comput 2022, 4, 63–74. 10.1007/s42514-021-00086-5. 35039800 PMC8754542

[ref39] Pihan E.; Kotev M.; Rabal O.; Beato C.; Diaz Gonzalez C. Fine Tuning for Success in Structure-Based Virtual Screening. J. Comput.-Aided Mol. Des. 2021, 35, 1195–1206. 10.1007/s10822-021-00431-4. 34799816

[ref40] Guo J.; Janet J. P.; Bauer M. R.; Nittinger E.; Giblin K. A.; Papadopoulos K.; Voronov A.; Patronov A.; Engkvist O.; Margreitter C. Dockstream: A Docking Wrapper to Enhance De Novo Molecular Design. J. Cheminf. 2021, 13, 89 10.1186/s13321-021-00563-7. PMC8596819 34789335

[ref41] Gadioli D.; Vitali E.; Ficarelli F.; Latini C.; Manelfi C.; Talarico C.; Silvano C.; Beccari A. R.; Palermo G. An Extreme-Scale Virtual Screening Platform for Drug Discovery. CF ‘22: Proceedings of the 19th ACM International Conference on Computing Frontiers 2022, 2022.

[ref42] Park H.; Lee J.; Lee S. Critical Assessment of the Automated Autodock as a New Docking Tool for Virtual Screening. Proteins 2006, 65, 549–554. 10.1002/prot.21183. 16988956

[ref43] Zhang S.; Kumar K.; Jiang X.; Wallqvist A.; Reifman J. Dovis: An Implementation for High-Throughput Virtual Screening Using Autodock. BMC Bioinf. 2008, 9, 126 10.1186/1471-2105-9-126. PMC2267697 18304355

[ref44] Trott O.; Olson A. J. Autodock Vina: Improving the Speed and Accuracy of Docking with a New Scoring Function, Efficient Optimization, and Multithreading. J. Comput. Chem. 2010, 31, 455–461. 10.1002/jcc.21334. 19499576 PMC3041641

[ref45] Gentile F.; Yaacoub J. C.; Gleave J.; Fernandez M.; Ton A. T.; Ban F.; Stern A.; Cherkasov A. Artificial Intelligence-Enabled Virtual Screening of Ultra-Large Chemical Libraries with Deep Docking. Nat. Protoc. 2022, 17, 672–697. 10.1038/s41596-021-00659-2. 35121854

[ref46] Yaacoub J. C.; Gleave J.; Gentile F.; Stern A.; Cherkasov A. Dd-Gui: A Graphical User Interface for Deep Learning-Accelerated Virtual Screening of Large Chemical Libraries (Deep Docking). Bioinformatics 2022, 38, 1146–1148. 10.1093/bioinformatics/btab771. 34788802

[ref47] Lu H.; Wei Z.; Wang C.; Guo J.; Zhou Y.; Wang Z.; Liu H. Redesigning Vina@Qnlm for Ultra-Large-Scale Molecular Docking and Screening on a Sunway Supercomputer. Front Chem. 2021, 9, 750325 10.3389/fchem.2021.750325. 34778205 PMC8581564

[ref48] Coleman R. G.; Carchia M.; Sterling T.; Irwin J. J.; Shoichet B. K. Ligand Pose and Orientational Sampling in Molecular Docking. PLoS One 2013, 8, e75992 10.1371/journal.pone.0075992. 24098414 PMC3787967

[ref49] Cannon B.; Smith N.; Stufft D. Pep 518—Specifying Minimum Build System Requirements for Python Projects. Python Enhancement Proposals, Pep 518, 2016. https://peps.python.org/pep-0518.

[ref50] Knight I. S.; Naprienko S.; Irwin J. J. Enrichment Score: A Better Quantitative Metric for Evaluating the Enrichment Capacity of Molecular Docking Models. arXiv 2022, arXiv:2210.10905v3 10.48550/arXiv.2210.10905.

[ref51] Meng E. C.; Gschwend D. A.; Blaney J. M.; Kuntz I. D. Orientational Sampling and Rigid-Body Minimization in Molecular Docking. Proteins 1993, 17, 266–278. 10.1002/prot.340170305. 8272425

[ref52] Reddy D. R. Speech Understanding Systems: A Summary of Results of the Five-Year Research Effort; Carnegie Mellon University, Department of Computer Science: Pittsburg, 1977.

[ref53] Mysinger M. M.; Shoichet B. K. Rapid Context-Dependent Ligand Desolvation in Molecular Docking. J. Chem. Inf. Model. 2010, 50, 1561–1573. 10.1021/ci100214a. 20735049

[ref54] Tingle B. I.; Irwin J. J. Large-Scale Docking in the Cloud. J. Chem. Inf. Model. 2023, 63, 2735–2741. 10.1021/acs.jcim.3c00031. 37071086 PMC10170500

[ref55] Lorber D. M.; Shoichet B. K. Fast, Hierarchical Ligand Docking. man. in prep, 2001.

[ref56] Lorber D. M.; Shoichet B. K. Hierarchical Docking of Databases of Multiple Ligand Conformations. Curr. Top. Med. Chem. 2005, 5, 739–749. 10.2174/1568026054637683. 16101414 PMC1364474

[ref57] Pettersen E. F.; Goddard T. D.; Huang C. C.; Couch G. S.; Greenblatt D. M.; Meng E. C.; Ferrin T. E. Ucsf Chimera-a Visualization System for Exploratory Research and Analysis. J. Comput. Chem. 2004, 25, 1605–1612. 10.1002/jcc.20084. 15264254

