# Supplementary material for: DockOpt: A Tool for Automatic Optimization of Docking Models
Source: J Chem Inf Model. 2024 Jan 11;64(3):1004–16. doi: 10.1021/acs.jcim.3c01406 (PMC10865354; doi:10.1021/acs.jcim.3c01406)
Supplement: Supplementary file 1 — ci3c01406_si_001.pdf [file ci3c01406_si_001.pdf]

Supporting Information for

DockOpt: A Tool for Automatic Optimization of Docking Models

Ian S. Knight, Olivier Mailhot, Khanh G. Tang, and John J. Irwin*

UCSF Department of Pharmaceutical Chemistry, 1700 4th St., San Francisco, CA 94158-2330

* Corresponding author: jji@cgl.ucsf.edu

Table of Contents

S1. Documentation about Pydock3, how to install it, and how to use its tools, including DockOpt and Blastermaster.

S2. Documentation about the DUDE-Z dataset toolkit, used to benchmark DockOpt against DUDE-Z.

S3. Empirical distributions of best normalized LogAUC for thin spheres radii parameters across DUDE-Z targets.

S4. Empirical poses of known ligands for HIVPR not included in DUDE-Z.

S5. Empirical distributions approximating the behavior of a truly random classifier in terms of normalized LogAUC.

S6. Cross-validation performance over all steps of beam search of four DUDE-Z targets that exhibited significant improvement in enrichment when transitioning from grid search to beam search.

S1. Documentation about Pydock3, how to install it, and how to use its tools, including DockOpt and Blastermaster.

https://github.com/docking-org/pydock3

S2. Documentation about the DUDE-Z dataset toolkit, used to benchmark DockOpt against DUDE-Z.

https://github.com/docking-org/dude-z-benchmark

S3. Empirical distributions of best normalized LogAUC for thin spheres radii parameters across DUDE-Z targets.

Here we present the mean across DUDE-Z target of the best enrichment witnessed for each combination of electrostatic and desolvation thin spheres radii, specifically for the models created using DockOpt with grid search.

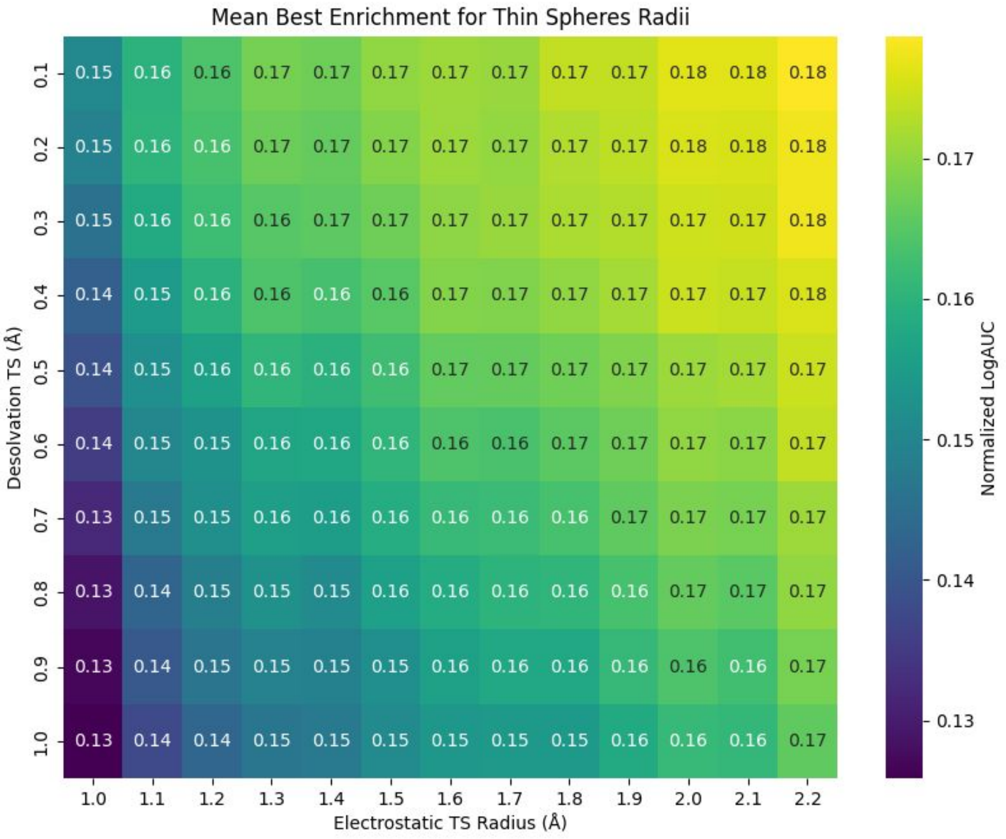

S4. Empirical poses of known ligands for HIVPR not included in DUDE-Z.

We obtained several known ligands for HIVPR not included in DUDE-Z to compare their poses to the poses predicted for the positive class molecules for HIVPR in DUDE-Z, with both groups being visualized in juxtaposition with the xtal-lig for HIVPR in DUDE-Z (PDB code: 1XL2).

pdb_code	raw_smiles	canonical_smiles
2HS1	CC(C)C[N@@](C[C@H]([C@H](Cc1ccccc1)NC(=O)O[C@H]2CO[C@@H]3[C@H]2CCO3)O)S(=O)(=O)c4ccc(cc4)N	CC(C)CN(C[C@@H](O)[C@H](Cc1ccccc1)NC(=O)O[C@H]1CO[C@H]2OCC[C@H]21)S(=O)(=O)c1ccc(N)cc1
2NMZ	CC(C)(C)NC(=O)[C@@H]1C[C@@H]2CCCC[C@@H]2C[N@]1C[C@H]([C@H](Cc3ccccc3)NC(=O)[C@H](CC(=O)N)NC(=O)c4ccc5ccccc5n4)O	CC(C)(C)NC(=O)[C@@H]1C[C@@H]2CCCC[C@@H]2CN1C[C@@H](O)[C@H](Cc1ccccc1)NC(=O)[C@H](CC(N)=O)NC(=O)c1ccc2ccccc2n1
6IXD	Cc1cc(cc(c1OCC(=O)N[C@@H](Cc2ccccc2)[C@@H](C(=O)N3CSC([C@H]3C(=O)N[C@H]4c5ccccc5C[C@H]4O)(C)C)O)C)NC(=O)CCCC[C@H]6[C@@H]7[C@H](CS6)NC(=O)N7	Cc1cc(NC(=O)CCCC[C@@H]2SC[C@@H]3NC(=O)N[C@@H]32)cc(C)c1OCC(=O)N[C@@H](Cc1ccccc1)[C@H](O)C(=O)N1CSC(C)(C)[C@H]1C(=O)N[C@H]1c2ccccc2C[C@H]1O
3DJK	CC(C)C[N@@](C[C@H]([C@H](Cc1ccccc1)NC(=O)O[C@@H]2CCOCOC2)O)S(=O)(=O)c3ccc(cc3)OC	COc1ccc(S(=O)(=O)N(CC(C)C)C[C@@H](O)[C@H](Cc2ccccc2)NC(=O)O[C@@H]2CCOCOC2)cc1
3FX5	CC(C)(C)NC(=O)[C@@H]1CSCN1C(=O)[C@H]([C@H](Cc2ccccc2)NC(=O)[C@H](CSC)NC(=O)COc3cccc4c3ccnc4)O	CSC[C@H](NC(=O)COc1cccc2cnccc12)C(=O)N[C@@H](Cc1ccccc1)[C@H](O)C(=O)N1CSC[C@H]1C(=O)NC(C)(C)C
5YOK	Cc1cccc(c1CNC(=O)[C@@H]2C(SCN2C(=O)[C@H]([C@H](Cc3ccccc3)NC(=O)[C@H]([C@H]4CCOC4)NC(=O)c5cc6cccc(c6o5)OC)O)(C)C)C	COc1cccc2cc(C(=O)N[C@H](C(=O)N[C@@H](Cc3ccccc3)[C@H](O)C(=O)N3CSC(C)(C)[C@H]3C(=O)NCc3c(C)cccc3C)[C@H]3CCOC3)oc12
3A2O	Cc1cc(cc(c1OCC(=O)N[C@@H](Cc2ccccc2)[C@@H](C(=O)N3CSC([C@H]3C(=O)NCC(=C)C)(C)C)O)C)N	C=C(C)CNC(=O)[C@H]1N(C(=O)[C@@H](O)[C@H](Cc2ccccc2)NC(=O)COc2c(C)cc(N)cc2C)CSC1(C)C

(1) Table of known ligands for HIVPR not included in DUDE-Z.

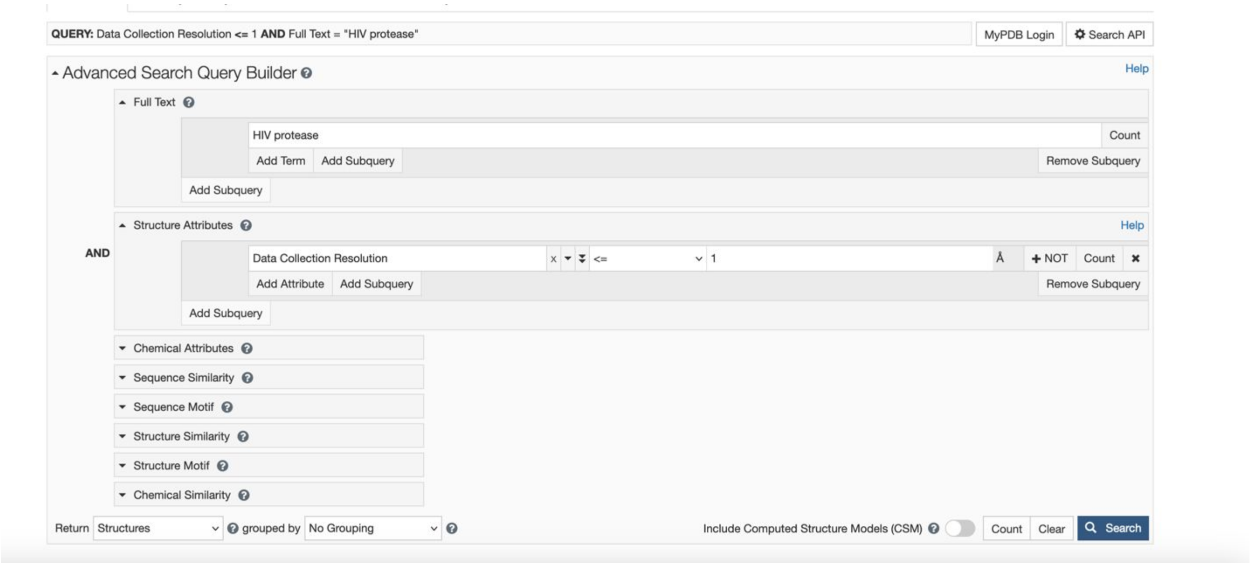

(2) PDB search query used to obtain the ligands in (1).

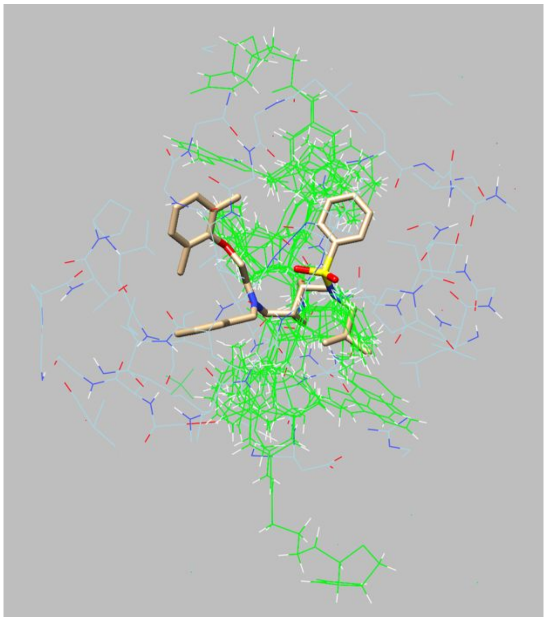

(3) Empirical poses of the ligands in (1) (green) juxtaposed with the empirical pose of the DUDE-Z xtal-lig for HIVPR (PDB code: 1XL2) (tan).

S5. Empirical distributions approximating the behavior of a truly random classifier in terms of normalized LogAUC.

The following plots show the distribution of normalized LogAUC calculated for different numbers of positives while holding a negative-to-positive ratio of 50:1 (as is the convention).

We performed 1 billion simulations for each number of positives, ranging from 1 to 100, for a total of 100 billion samples taken. Here we show (1) the empirical distributions for the number of positives ranging from 1 to 50, and (2) how the p-value to distinguish observed normalized LogAUC from the empirical distribution behaves with respect to the number of positives (AKA "actives"). The empirical distributions are binned using a width of 0.1%.

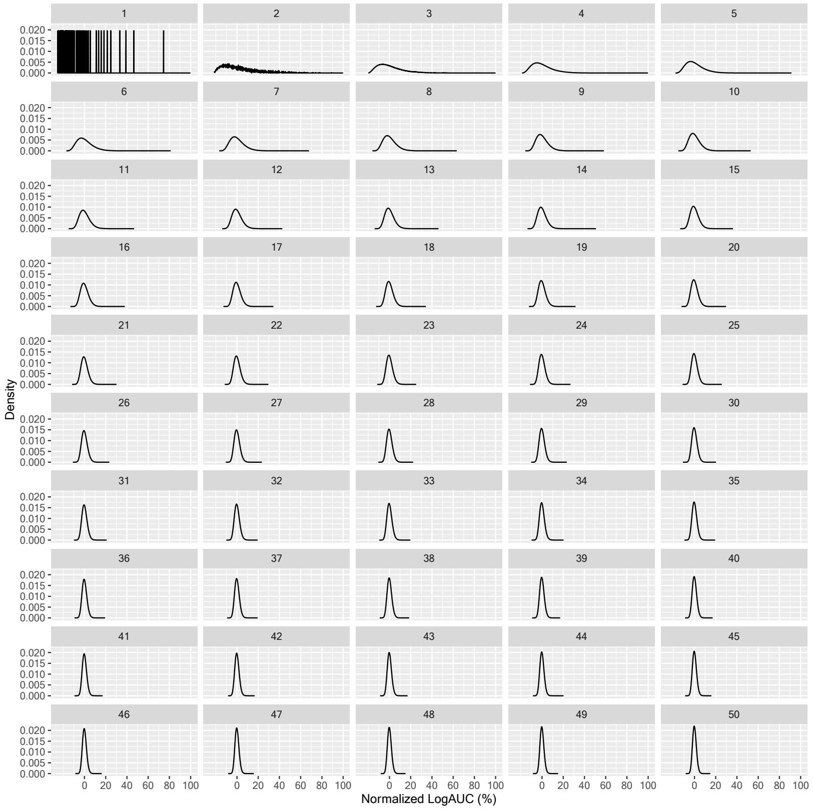

(1) The empirical distributions for the number of positives ranging from 1 to 50

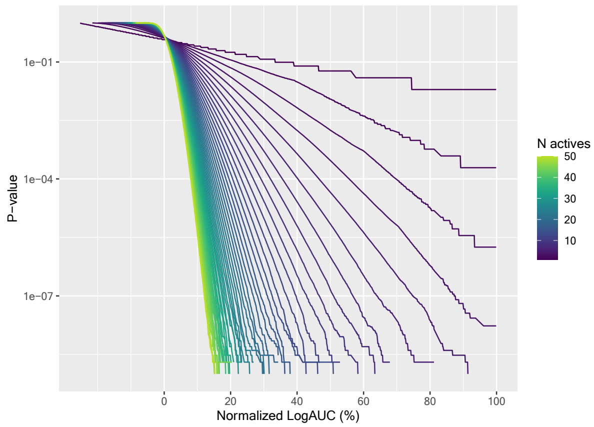

(2) How the p-value to distinguish observed normalized LogAUC from the empirical distribution behaves with respect to the number of positives (AKA “actives”).

S6. Cross-validation performance over all steps of beam search of four DUDE-Z targets that exhibited significant improvement in enrichment when transitioning from grid search to beam search.

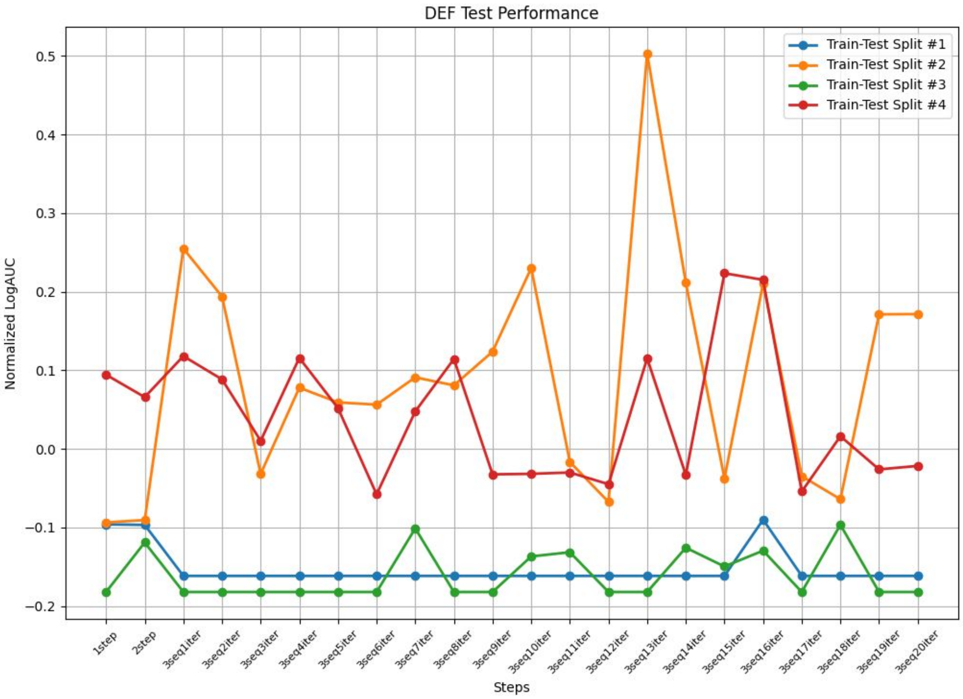

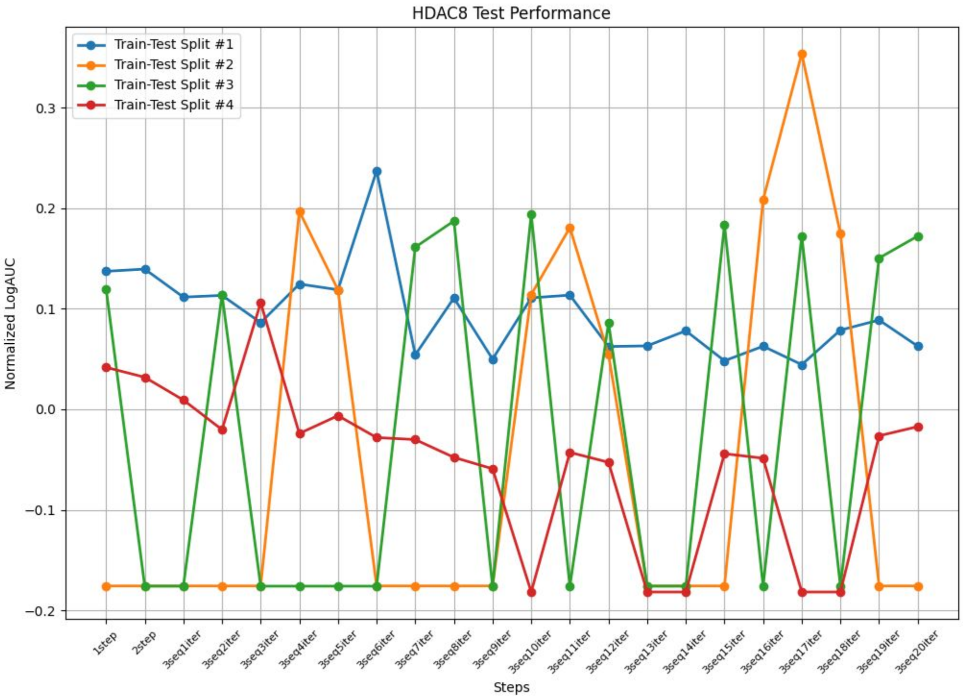

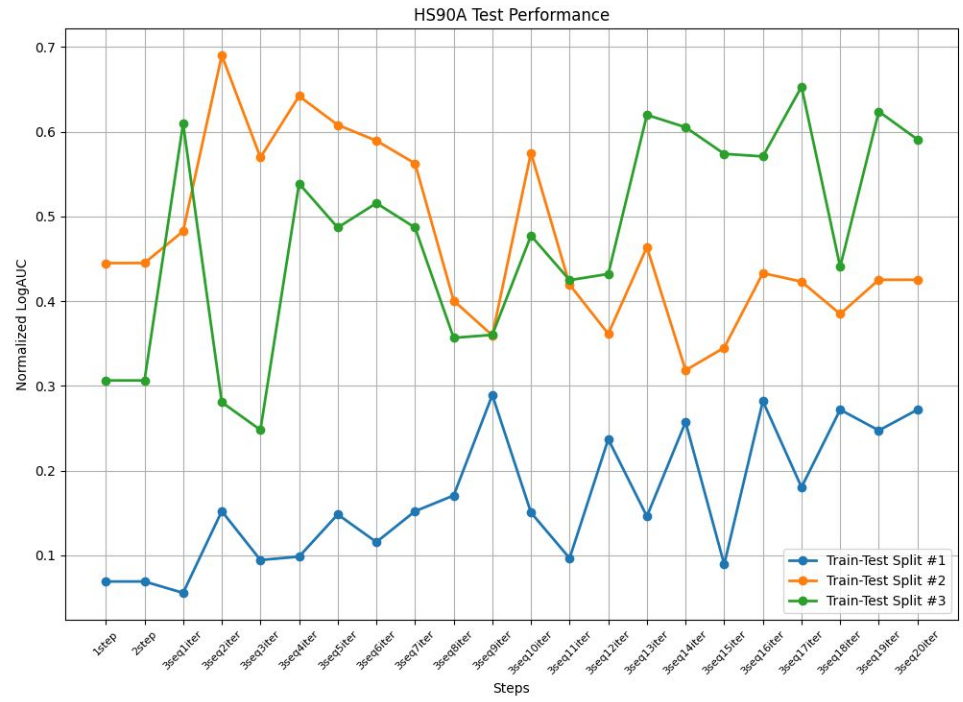
HS90A Test Performance
Normalized LogAUC
Steps
Train-Test Split #1
Train-Test Split #2
Train-Test Split #3
1step
2step
3seq1iter
3seq2iter
3seq3iter
3seq4iter
3seq5iter
3seq6iter
3seq7iter
3seq8iter
3seq9iter
3seq10iter
3seq11iter
3seq12iter
3seq13iter
3seq14iter
3seq15iter
3seq16iter
3seq17iter
3seq18iter
3seq19iter
3seq20iter

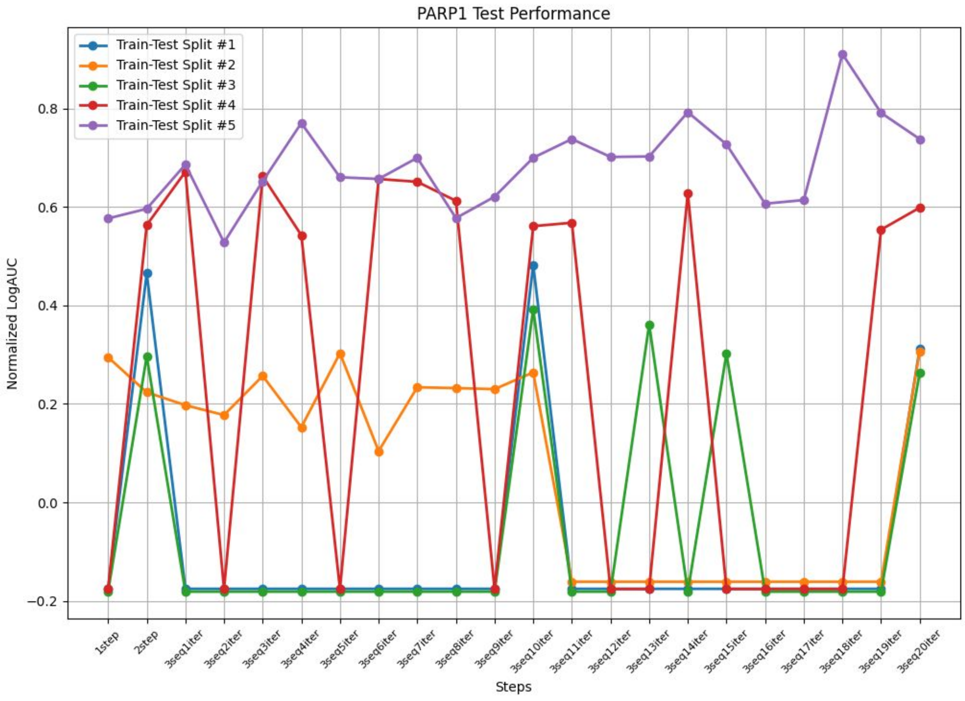
PARP1 Test Performance
Normalized LogAUC
Steps
Train-Test Split #1
Train-Test Split #2
Train-Test Split #3
Train-Test Split #4
Train-Test Split #5
1step
2step
3seq1iter
3seq2iter
3seq3iter
3seq4iter
3seq5iter
3seq6iter
3seq7iter
3seq8iter
3seq9iter
3seq10iter
3seq11iter
3seq12iter
3seq13iter
3seq14iter
3seq15iter
3seq16iter
3seq17iter
3seq18iter
3seq19iter
3seq20iter
